# Understanding why consumers in China switch between wild, farmed, and synthetic bear bile products

**DOI:** 10.1111/cobi.13895

**Published:** 2022-04-29

**Authors:** Amy Hinsley, Anita Kar Yan Wan, David Garshelis, Michael Hoffmann, Sifan Hu, Tien Ming Lee, Keila Meginnis, Brendan Moyle, Yingjie Qiu, Xiangdong Ruan, E. J. Milner‐Gulland

**Affiliations:** ^1^ Department of Zoology University of Oxford Oxford UK; ^2^ School of Life Sciences and Ecology and State Key Laboratory of Biological Control Sun Yat‐Sen University Guangzhou People's Republic of China; ^3^ IUCN SSC Bear Specialist Group Cohasset Minnesota USA; ^4^ Conservation Programmes Zoological Society of London London UK; ^5^ IUCN Species Survival Commission International Union for Conservation of Nature Gland Switzerland; ^6^ Institute of Health and Wellbeing University of Glasgow Glasgow UK; ^7^ School of Economics and Finance Massey University Palmerston North New Zealand; ^8^ China Association of Traditional Chinese Medicine Beijing People's Republic of China; ^9^ Academy of Inventory and Planning National Forestry and Grassland Administration Beijing People's Republic of China

**Keywords:** Asiatic black bear, consumer behavior, consumer demand, stated preferences, supply‐side approaches, wildlife farming, comportamiento del consumidor, crianza de fauna, demanda del consumidor, estrategias del lado del suministro, oso negro asiático, preferencias manifestadas, 亚洲黑熊;消费需求;消费行为;陈述性偏好;供应端方法;野生动物饲养

## Abstract

An important rationale for legally farmed and synthetic wildlife products is that they reduce illegal, wild‐sourced trade by supplying markets with sustainable alternatives. For this to work, more established illegal‐product consumers must switch to legal alternatives than new legal‐product consumers switch to illegal wild products. Despite the widespread debate on the magnitude and direction of switching, studies among actual consumers are lacking. We used an anonymous online survey of 1421 traditional Chinese medicine consumers in China to investigate switching among legal farmed, synthetic, and illegal wild bear bile. We examined the past consumption behavior, applied a discrete choice experiment framed within worsening hypothetical disease scenarios, and used latent class models to investigate groups with shared preferences. Bear bile consumers (86% respondents) were wealthier, more likely to have family who consumed bile, and less knowledgeable about bile treatments than nonconsumers. Consumer preferences were heterogenous, but most consumer preferences switched between bile types as disease worsened. We identified five distinct latent classes within our sample: law‐abiding consumers (34% respondents), who prefer legal products and were unlikely to switch; two all‐natural consumer groups (53%), who dislike synthetics but may switch between farmed and wild; and two nonconsumer groups (12%), who prefer not to buy bile. People with past experience of bile consumption had different preferences than those without. Willingness to switch to wild products was related to believing they were legal, although the likelihood of switching was mediated by preferences for cheaper products sold in legal, familiar places. We found that consumers of wild bile may switch to legal alternatives, given the availability of a range of products, whereas legal‐product consumers may switch to illegal products if the barriers to doing so are small. Understanding preferences that promote or impede switching should be a key consideration when attempting to predict consumer behavior in complex wildlife markets.

## Abstract 2

*Article Impact Statement*: Wildlife consumer characteristics and preferences determine their likelihood and direction of switching between legal and illegal products.

## Abstract (es)

La Importancia de Comprender Por Qué los Consumidores en China Cambian entre Productos de Bilis de Oso Silvestre, Criado y Productos Sintéticos

**Resumen:**

Una razón importante detrás de los productos faunísticos de crianza legal y los productos sintéticos es que reducen el mercado ilegal de origen silvestre al proporcionarles alternativas sustentables a los mercados. Para que esto funcione, deben ser más los consumidores establecidos de productos ilegales que cambian a las alternativas legales que los consumidores nuevos de productos legales que cambian a productos silvestres ilegales. A pesar del debate extenso sobre la magnitud y dirección del cambio, los estudios sobre consumidores reales son deficientes. Encuestamos en línea a 1421 consumidores anónimos de medicina tradicional china en ese país para investigar los cambios entre bilis de oso silvestre e ilegal, sintética y criada legalmente. Examinamos el comportamiento pasado de los consumidores, aplicamos un experimento de elección discreta enmarcado dentro de escenarios de agudizamiento de enfermedades, y usamos modelos latentes de clase para investigar a los grupos con preferencias compartidas. Los consumidores de bilis de oso (86% de los respondientes) resultaron ser más adinerados, con mayor probabilidad de tener familiares que consumen bilis y menos conocedores sobre los tratamientos con bilis que los no consumidores. Las preferencias de los consumidores fueron heterogéneas, aunque la mayoría de éstas cambiaba de tipo de bilis conforme la enfermedad empeoraba. Identificamos cinco clases latentes distintivas dentro de nuestra muestra: los consumidores que respetan las leyes (34% de los respondientes), quienes prefieren los productos legales y probablemente no cambiarían de tipo de producto; dos grupos de consumidores de productos naturales (53%), quienes no gustan de los productos sintéticos, pero podrían cambiar entre aquellos de crianza y silvestres; y dos grupos de no consumidores (12%), quienes prefieren no comprar bilis. Las personas con experiencia previa en el consumo de bilis tuvieron preferencias diferentes a aquellas sin experiencia. La voluntad por cambiarse a productos de origen silvestre estuvo relacionada con la creencia de que fueran legales, aunque la probabilidad de cambiar estuvo mediada por la preferencia por productos más baratos vendidos en lugares legales y conocidos. Descubrimos que los consumidores de bilis silvestre pueden cambiar a las alternativas legales dada la disponibilidad de una gama de productos, mientras que los consumidores de productos legales pueden cambiar a los productos ilegales si las barreras para hacerlo son menores. El entendimiento de las preferencias que promueven o impiden estos cambios debería ser una consideración importante cuando se intente predecir el comportamiento del consumidor dentro de los mercados complejos de fauna.

## 摘要

了解中国消费者在野生、饲养和合成的含熊胆药品/制品之间“转换”的原因

对于合法饲养和合成的野生动物产品而言, 一个重要的原因在于其可通过为市场提供可持续的替代品来减少非法野生来源的贸易。为实现这一点, 转向合法替代品的固有非法产品消费者必须多于新增的转向非法野生产品的合法产品消费者。尽管关于转换的规模与方向有着广泛的讨论, 但仍缺乏对实际消费者的研究。我们通过对中国 1421 名中药消费者的在线匿名调查来探究在合法饲养、合成和非法野生的含熊胆药品/制品之间存在的转换。我们调查了过去的消费行为, 同时在假设的疾病逐步恶化的情景中应用了离散选择实验, 并使用潜在类别模型来研究具有共同偏好群体的特征。含熊胆药品/制品的消费者(86% 的受访者)与非消费者相比经济条件更优、更有可能其家人有过此类消费, 并且对含熊胆药品/制品的治疗方面的了解更少。消费者的偏好是异质的, 但随着疾病的恶化, 大多数消费者更倾向于在不同类型的含熊胆药品/制品之间进行转换。我们在样本中确定了五个不同的潜在人群类别:“守法消费者”(34% 的受访者), 他们更喜欢合法产品并且不太可能转换;两个“纯天然消费者”群体(53%), 他们不喜欢合成的产品, 但可能会在饲养和野生的产品之间转换;以及两个不喜欢购买含熊胆药品/制品的“非消费者”群体(12%)。有过含熊胆药品/制品消费经历的群体与没有消费过的人群有着不同的偏好。尽管转换的可能性会受到对在合法、熟悉地方销售的低价产品偏好的调节影响, 但转向野生产品的意愿与认为其是合法的有关。我们的研究表明, 考虑到一系列合法替代品的可获得性, 野生熊胆的消费者可能会转向合法产品, 而如果转换的阻碍很小, 原本合法产品的消费者也可能会转向非法产品。在复杂的野生动物市场中尝试预测消费者的行为时, 了解可促进或阻碍转换的偏好特征是一个需要考虑的至关重要因素。

## INTRODUCTION

Understanding consumer behavior is an important step toward designing effective interventions to reduce the illegal wildlife trade. This is a particular priority when designing interventions that aim to encourage consumers to switch from wild products, where demand may threaten species persistence if off‐take is unsustainable, to a farmed, synthetic, or an alternative species with the aim of providing sustainability (Thomas‐Walters et al., 2020). Farmed versions of an original wild product have increased the diversity of a number of wildlife markets, such as ornamental plants (e.g., orchids [Hinsley et al., 2015]), wild meat (e.g., turtles [Nuno et al., 2018], porcupines [Brooks et al., 2010]), and traditional medicines (e.g., bear bile [Dutton et al., 2011]). There are also examples of synthetic alternatives or those derived from other wild or domesticated species, including furs and skins (e.g., synthetic leopard skins [Naude et al., 2020]) and medicines (e.g., lion bone or herbal alternatives for tiger bone [Moorhouse et al., 2020]). For these supply‐side approaches to be effective at reducing demand for wild products, a large proportion of consumers of wild products must switch to the alternatives when they become available (Mi et al., 2019). These approaches may backfire if the availability of legal alternatives reduces the social stigma of consumption and leads to increased use of illegal products (Rizzolo, 2021). To evaluate the potential benefits and prevent unintended consequences of these interventions, it is vital to understand the magnitude, direction, and motivation for switching between legal and illegal products specific to each market. This is particularly important now because farmed, synthetic, or other legal alternatives are being proposed as strategies to reduce illegal trade in several high‐profile species (e.g., rhinoceros horn [Mi et al., 2019]).

Switching behavior can be challenging to fully comprehend, due to the complexity of wildlife markets and the nuances of consumer behavior and the factors that influence it. Several studies of wildlife markets with legal and illegal alternatives report that farming does not effectively reduce demand for preferred wild‐sourced products (e.g., bear bile [Dutton et al., 2011], turtle meat, or eggs [Nuno et al., 2018]). Although fewer studies of synthetic alternatives exist, some report preferences for (e.g., wildlife‐based medicines [Davis et al., 2016; Liu et al., 2016]) and uptake of synthetic alternatives (Naude et al., 2020). Furthermore, interest in the use of herbal over animal‐based products has been found (Moorhouse et al., 2020). However, while researchers have drawn conclusions about switching, there are often limitations in the applicability of their findings to actual consumer behavior. First, wildlife consumers can be difficult to access, meaning that many conclusions about probable use of alternative products are based on samples dominated by nonconsumers (e.g., <20% sample were consumers [Dutton et al., 2011], <30% were consumers [Liu et al., 2016]). Second, people may try to conceal their behavior, especially if it pertains to the consumption of an illegal or socially sensitive product (Nuno et al., 2018). Furthermore, many researchers assumed that consumer choice is driven solely by the source (e.g., wild, farmed, or synthetic [Hinsley & ‘t Sas‐Rolfes, 2020]), even though wildlife markets are likely to be much more complex, with multiple factors affecting consumer decisions (e.g., Hanley et al., 2017). A more nuanced and rigorous approach to consumer behavior is needed to design and evaluate these interventions (Thomas‐Walters et al., 2020).

We investigated preferences that could underpin switching behavior among self‐reported consumers of wildlife products. We focused on consumers of traditional Chinese medicine (TCM) in mainland China, and framed our questions around the consumption of bear bile, a TCM product used since at least AD 649 (Feng et al., 2009). In China, bear bile is most commonly used as a treatment for ailments of the liver and eyes and a cooling medicine for *heatiness*, a term for a general feeling of being unwell. It is legally sourced from farms, although illegal markets for wild bile exist (Hinsley et al., 2021). The only known active ingredient in bear bile, ursodeoxycholic acid (UDCA), is used in biomedicine (e.g., the United Kingdom's recommended treatment for primary biliary cirrhosis [NHS, 2019]) and sold and prescribed as a synthetic product worldwide, often in the form of a capsule. Although current consumers of bear bile in Chinese markets can buy farmed, wild, or synthetic products, this was not always the case.

Historically, bear bile was obtained only from the gallbladders of killed wild bears (principally Asiatic black bears [*Ursus thibetanus*]), but the species’ rarity made the product increasingly expensive and difficult to obtain. In the 1970s, North Korea developed a method of extracting bile from live, captive bears, which was adopted in China in the early 1980s (Feng et al., 2009). At the same time as legal farming began, China implemented legislation that made the hunting and trade of wild bear bile illegal (e.g., the 1989 Wild Animal Protection Law) (Huang & Li, 2007). This raised the key question as to how the vast supply of cheap, legal, and easily available farmed bear bile affected the demand for wild bile, which today remains expensive, illegal, and much more difficult to procure. With wild bile often viewed as more efficacious (Hinsley et al., 2021), were more past or potential wild bile users satisfied with using only farmed bile or did a larger number of new consumers, drawn into the market by farmed bile, use wild bile on occasion? What matters for bear conservation is not how many people switch but the number of wild gallbladders consumed and whether the number of bears killed for them is sustainable. Illegal killing of Asiatic black bears is considered the most severe threat to this globally vulnerable species (Garshelis & Steinmetz, 2020).

In addition to the complexity related to consumer behavior, attempts to understand broader relationships between wildlife farming and demand for wild products are often confounded by other serious issues, which include animal welfare and human health, the latter having gained increasing attention following the COVID‐19 pandemic (Watsa et al., 2020). For bear farming, animal welfare concerns related to the small cages in which bears were kept, frequent infections, low survival, and poor breeding, which necessitated restocking from the wild, led to the Chinese government to impose greater restrictions on the industry (Huang & Li, 2007). Under current regulations, bears cannot be confined to small cages or have an external catheter, and there must be a minimum of 200 bears kept on farms (NFGA, 2017). Further complicating the situation, Chinese bear bile markets are not independent of those in other countries where bear bile demand also exists. Illegal trade occurs in both directions between Southeast Asia and China (e.g., Laos [Gomez & Shepherd, 2018]). Although bear bile from China cannot be legally exported, illegal farms exist in many Southeast Asian countries, and they are often stocked with wild bears (e.g., Vietnam [Crudge et al., 2020]), presenting a direct threat to wild populations.

The question of whether bear or other wildlife farming has reduced pressure on wild species has been the subject of much debate, with a lack of evidence in many markets posing a challenge for conservation decision makers. For bear farming, this was recognized by a formal recommendation adopted by government and nongovernmental members at the 2012 International Union for Conservation of Nature's (IUCN) World Conservation Congress. The recommendation calls for a situation analysis of relationships between bear farming and conservation of wild bears (portals.iucn.org/library/node/44106). China provides an interesting case study to examine these relationships because consumers in Chinese markets differ from other bear bile consumers by having widespread access to legal farmed and synthetic UDCA and some access to illegal wild bear bile products. That these products are part of official patent‐medicine TCM (farmed), informal Chinese medicine that is not TCM‐sanctioned (wild), and biomedicine (synthetic UDCA) means a case study of switches between these products would show how people use medicines from different systems (medical pluralism). Furthermore, these products also differ with regard to their form (raw vs. processed), place of purchase (formal vs. informal), and other attributes.

To investigate consumer preferences in this complex market and to draw wider conclusions on factors that influence wildlife consumers to switch between wild products and legal alternatives, we used a discrete choice experiment (DCE). The DCE is a well‐established method designed to elicit consumer preferences for nonmarket goods (i.e., goods for which real market price and demand data are unavailable) (Johnston et al., 2017). The method has been used in several environmental contexts, including studies of legal and illegal wildlife consumption (Hanley et al, 2017; Hinsley et al., 2015). DCEs have been used to study Chinese bear bile markets, but unrealistic scenarios were used to inquire about bear bile (e.g., considering wild bile as legal) or the samples were dominated by respondents who had never heard of bile, rendering the results largely hypothetical (Dutton et al., 2011; Liu et al., 2016). Our aim was to reduce hypothetical bias within a stated preference study by sampling self‐reported consumers and designing our DCE to reflect real‐world market conditions as much as possible.

Studies of the wider public in China suggest that most people have never heard of bear bile, let alone consumed it, and that consumers and nonconsumers have different characteristics (Hinsley et al., 2021). We, therefore, hypothesized that previous exposure to a product affects switching behavior, so we applied our DCE to a targeted sample of TCM consumers who had heard of bear bile. We also asked direct questions prior to the DCE about personal consumption of different bile types to examine preferences and switching associated with past consumption experience. Furthermore, whereas many studies of wildlife consumption focus on preferences relating to the wild, farmed, or synthetic source of a product (Hinsley & ‘t Sas‐Rolfes, 2020), we hypothesized that purchasing behavior is affected by a broad range of contextual factors. We, therefore, included DCE attributes, such as place of purchase and product form. Finally, based on significant heterogeneity of consumer preferences in wildlife markets and the likelihood that preferences not only vary among demographic groups (Hinsley et al., 2015), but also change as health deteriorates (Dutton et al., 2011), we hypothesized that preferences are complex and circumstance dependent. We, therefore, applied a latent class modeling (LCM) approach to our sample, which accounts for individual heterogeneity by estimating a finite set of discrete classes with differing preference profiles across classes; it estimates whether individual characteristics significantly predict class membership and links different preferences to individual characteristics (Greene & Hensher, 2003). Furthermore, we used progressively worsening disease scenarios to look at how these affect preferences in different classes. Overall, we aimed to provide in‐depth data on the factors influencing consumer behavior and draw conclusions on the situations in which consumers may switch in different directions between legal and illegal wildlife products.

## METHODS

We implemented an online DCE with a targeted sample composed of residents of mainland China who were over 18 years old, had heard of bear bile, and reported using TCM. Our aim was to reach a large and diverse sample of current and potential bear bile consumers (Appendix S1). Although online surveys may produce a less representative sample of the public than face‐to‐face surveys, they are particularly useful for accessing difficult‐to‐reach groups. This was important because previous work on Chinese bear bile markets shows that face‐to‐face surveys find relatively few actual consumers (e.g., Dutton et al., 2011; Hinsley et al., 2021). We also chose to use an anonymous online survey because they improve honesty in reporting of sensitive and illegal behavior compared with face‐to‐face surveys (Kreuter et al., 2008). We reduced the risk of fake answers by using a panel, rather than sending out survey links publicly, and employing a multistep quality control process to produce our final sample (Appendix S1).

For one month (July–August 2019), we sent out single‐use survey links to randomly selected respondents from a nationwide panel of 2.6 million members on the Wen Juan Xing (WJX, 问卷星 www.wjx.cn/) survey platform. Respondents who completed the survey received a small credit (<10 CNY) to their WJX account.

All research was approved by the University of Oxford's Central University Research Ethics Committee (approval reference: R55074/RE001) and by the China Association of TCM, Sun Yat Sen University, and the Academy of Inventory and Planning at the National Forestry and Grassland Administration in China.

### Piloting

We designed a simple orthogonal DCE in SPSS 25 (IBM, 2017) that presented choice sets that included product price, source, form, place of purchase, availability, and recommendation by different people. We piloted this in face‐to‐face surveys with 32 members of the public in Guangzhou in March 2018 in which we asked respondents to select their preferred product and discuss the attributes influencing their decision. We made some changes based on these results. Specifically, we combined the availability and place‐of‐purchase attributes, added more information about disease type and severity, and framed choices in the context of a doctor suggesting bear bile as a treatment. The latter decision was made because all respondents stated that they would want to first check with a doctor that bear bile was suitable for their ailment. We then piloted the survey online with 162 randomly selected WJX panel members (including 59 bile consumers), resulting in small improvements to clarify the DCE instructions (Appendix S2).

### Final survey

**FIGURE 1:**
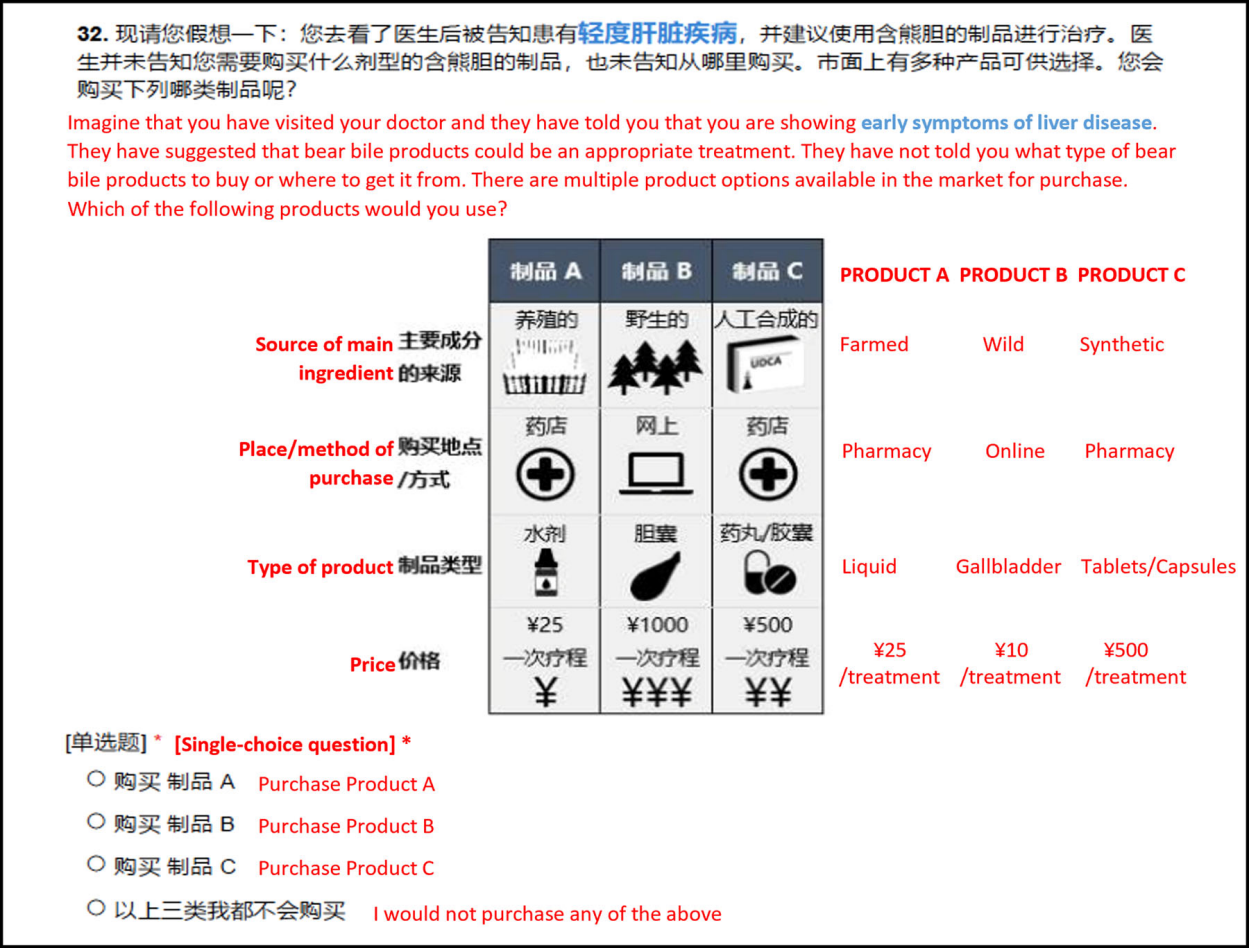
An example of final choice questions (presented to respondent only in Chinese, annotated here with an English translation) in an online survey on bear bile consumption and consumer preferences in China, showing the framing statement, scenario, choice card, and no choice option

We used the bile consumer data from our second pilot to provide priors for a D‐efficient Bayesian design with four attributes: type (wild, farmed, and synthetic); form (gallbladder, tablet or capsules, powder, tea, medicinal wine, and other liquids); place of purchase (pharmacy, hospital, TCM market, online, personal contact, and bear farm); and price per course of treatment in Chinese Yuan (CNY) (25, 250, 500, 1000, 2500, and 5000) (Figure 1). We also included three disease scenarios, coded as attributes in the design but presented as part of the question framing (as recommended by Choicemetrics, 2018). These scenarios progressed from mild (eye infection) to intermediate (early‐stage liver disease) to severe (advanced liver disease) conditions, and we ordered our choice sets by scenario to create this progression (i.e., respondents saw choices in three mild, then three intermediate, and then three severe scenarios). This provided a specific motivation for choice while allowing us to look at whether preferences changed as disease worsened. We used Ngene 1.2 (ChoiceMetrics, Sydney, Australia) to produce a final design with 500 Sobol draws from a normal distribution for each parameter prior (Bliemer et al., 2008). After >90,000 evaluations, we selected the design with the lowest D error (0.39); it had 18 choice sets of three alternatives. We randomly assigned respondents to one of two blocks of nine of these choice sets. Each choice was framed as a personal choice following a doctor's suggestion that bear bile was a potential treatment for the ailment specified. Recognizing that TCM consumers may have poor knowledge of different products (Liu et al., 2016), we used unlabeled alternatives (e.g., product 1) rather than labeled (e.g., product names) because they are suited to studies of how consumers with poorer product knowledge trade‐off between different attributes to make choices (De Bekker‐Grob et al., 2010). We also provided an annotated practice choice question and required respondents to confirm their understanding of the definitions of *wild*, *farmed*, and *synthetic bile* to reduce confusion about source.

The results of stated preference methods are not perfect representations of real behavior, so we took several steps to reduce hypothetical bias and ensure that respondents answered questions as close as possible to the way they would act in a real‐life situation. First, we used published literature, pilot studies, and author observations to determine attribute combinations that best‐reflected real market environments in China (Appendix S3). For example, respondents would not see a choice set with synthetic bile sold as a gallbladder, but might see wild bile sold in a pharmacy; we observed this in very rare cases. Second, we provided an opt out in each choice set so as not force respondents to select a product they would not choose in real life. Respondents who opted out could state whether they would prefer to use a different bear bile product, another animal bile or herbal product, a biomedicine, or to consult another (TCM or biomedical) doctor. Finally, we asked respondents to swear a “solemn oath” that they would answer as if it was a real‐life situation. This technique improves honesty in stated preference studies (Jacquemet et al., 2017). We also emphasized at the start that the results would be used by international and national‐level decision makers to encourage people to take their choices more seriously.

We asked questions about personal consumption of bear bile from different sources, as well as several demographic, knowledge, behavior, and perception questions designed to determine characteristics associated with bile consumption and to provide covariates for LCMs (Appendix S4). For example, we asked when the person had last visited a biomedical doctor and a TCM doctor. There was also a time‐limited knowledge question, asking respondents to name, in 60 s, the ailments that bear bile could be used to treat from a list containing three real uses (liver disease, eye problems, and heatiness) and three false non‐TCM‐sanctioned uses (kidney disease, boosting calcium, and improving brain power). The false uses were chosen by TCM experts in our author team and checked to ensure they were not TCM‐sanctioned uses for bear bile listed in the official *Pharmacopoeia* (National Pharmacopeia Committee, 2015). They were also not reported by any practitioners as a use for bear bile in a related study of bear bile use and prescription in China (Hinsley et al., 2021). We also included two control preference scale (CPS) questions asking the degree of control respondents prefer in treatment decisions (Degner et al., 1997) for mild (eye infection) and severe (liver disease) ailments, from fully active (“I prefer to make all decisions regarding treatment.”) to fully passive (“I prefer to leave all decisions regarding treatment to my doctor.”).

### Analyses

We calculated descriptive statistics and fitted model‐averaged generalized linear models (GLMs); past bear bile consumption by the respondent was the dependent variable. Our independent variables were selected based on the published literature (Appendix S5) and covered demographics (age, income, gender, and resident in province with bear farms), a knowledge test on bile use (respondents passed if they named only real TCM‐sanctioned uses), knowledge of legality and perceived conservation impacts of wild bile trade (wild bile legality and wild bear population status), exposure to consumption (having family members or friends or colleagues who use bile), and preferred role in treatment decisions (CPS score for mild and severe disease). We tested for collinearity between covariates before running models with a Cramer's *V* test. Respondents could report the consumption of multiple types of bile, so we ran one GLM for each of wild, farmed, synthetic, and all types combined (i.e., had consumed at least one type) in the R package Mumin’ 1.43.6 (Barton, 2019; R Core Team, 2019). All models with ΔAIC (Akaike information criterion) <4 were averaged. Many consumers gave inconsistent answers on the source and form of bile used, so we ran an additional GLM for higher‐certainty wild bile consumption, which included people who reported consuming both wild bile and gallbladders or wine, which are the forms most likely to be wild sourced.

We began our estimations of preferences with multinomial and mixed logit models. To examine heterogeneity more closely, we estimated LCMs with 2–7 classes with Latent Gold 5.1 (Statistical innovations, 2019), which outperformed our multinomial and mixed logit models based on standard information criteria. The LCMs grouped consumers into “latent classes” depending on shared preferences (Greene & Hensher, 2003) (Appendix S6). To better understand characteristics of consumers in each class, we used the same covariates as in the bile‐consumption GLMs plus three dummy covariates of past consumption of farmed, wild, and synthetic bile, which refined the models based on significance of covariates in predicting membership. We then categorized each class by consumer type to clearly show how they could potentially be used in the future to target interventions.

We examined how medical condition scenario (eye infection, early‐stage and severe liver disease) interacted with various attributes to determine whether different preferences existed in the different framing scenarios. The final set of interactions was selected based on variable testing and consisted of interactions of two scenario dummies (early‐stage liver disease and severe liver disease) with price and product type (farmed and synthetic). We identified the final number of LCM classes with Bayesian information criterion.

Finally, we calculated willingness to pay (WTP) in Latent Gold, which uses the Delta method for estimating standard errors (Hole, 2007). The WTP is a measure that shows how much respondents would pay in CNY to secure treatment with a product that had the targeted attribute level when compared with the base level (e.g., to secure farmed over wild bile).

## RESULTS

We sent out 18,865 survey links and received 1843 complete responses of which 1421 passed all quality control tests. This final sample had a broad range of respondents, although it had a higher proportion of women (59.6%) younger (85.2% <45 years) and educated people (>90% with undergraduate degrees) than the national average (www.stats.gov.cn) (Appendix S7). Respondents were from 29 provinces, municipalities, and autonomous regions across China. The regions with the largest numbers of respondents were the east (*n* = 460), followed by south‐central (*n* = 446), north (*n* = 241), and southwest (*n* = 136) China.

Due to our targeted sampling strategy, 90.4% of the final sample had visited a TCM practitioner in the past year, and most had also visited a biomedical practitioner in this time (84.9% sample). In addition, 56.2% had consumed bear bile in the prior 12 months, and the majority (85.6%) had bought or used bile in their lifetime. Slightly more (90.7%) said they would use it in the future. Most consumers reported using bile for medicine (71.8% of 1421). Furthermore, 79.8% of respondents knew at least one bile consumer, most frequently parents (40.0% respondents) and grandparents (32.8%). Only 3.3% of respondents had never used bile, knew no consumers, and would not use bile in the future.

The most frequently selected option in the CPS for severe disease was the most passive (“I prefer to leave all decisions regarding treatment to my doctor.”), whereas for mild disease, it was the second most active (“I prefer to make the final decision about treatment after seriously considering my doctor's opinion.”). Although 97.0% of the sample named true TCM‐sanctioned uses of bile in the treatment‐knowledge question, 507 also selected at least one false non‐TCM‐sanctioned use. Therefore, 61.3% of respondents passed the knowledge test. In addition, 64.5% respondents correctly indicated that wild bile was illegal, and 82.9% believed that wild bear populations in China were decreasing (current bear population trends in China are unknown).

More than half of respondents reported past consumption of farmed bile (56.2%) or synthetic bile (53.3%), whereas 16.7% had used wild bile. The most frequently consumed product forms were patent products (e.g., eye drops: 37.8%), which are likely to be from farmed or synthetic sources, although 18.1% of the sample reported having used a bear gallbladder, a raw form likely to be wild sourced in China (although fake gallbladders are also sold). Mismatches between reported wild bile and gallbladder consumption confirm that consumers may not know the true source of products they use. Our higher‐certainty wild bile category was, therefore, used to provide an estimate that 11.0% of the sample (*n* = 156) is most likely to have used wild bile. More than half of respondents had bought bile in hospitals (53.8%) and pharmacies (59.1%), with the fewest reporting purchasing from personal contacts (7.3%) or bear farms (6.5%) (Appendix S8).

In our GLMs, at 95% confidence intervals, men, and people who knew other bile consumers, were more likely to report bile consumption overall (Table 1). Consumers of specific bile types had broadly similar characteristics, such as being in higher income categories, having family members who consume bile, and being less likely to pass the knowledge test on treatment uses. The latter meant that nonconsumers were more likely to select only TCM‐sanctioned uses of bile. When comparing consumers of different bile types, consumers of wild, farmed, and synthetic bile were more likely to be 26–45 years. Farmed bile consumers were more likely to have friends who consumed bile. Men were more likely to consume farmed, wild, and higher‐certainty wild bile than women. All wild bile consumers were more likely to state that wild bile was not illegal. Living in a province with a bear farm and CPS for severe disease were not associated with any consumption type, whereas perception of bear status and mild disease CPS answers were only significantly associated with consumption at 90% confidence intervals.

**TABLE 1:** Summary of model‐averaged results for five generalized linear models of characteristics associated with bear bile consumption with type of bile consumed as the dependent variable (*n*=1395)^a^

			Specific bile type consumed estimate (SE)^b^			
Covariate		Consumed any bile estimate (SE)^b^	higher‐certainty wild	wild	farmed	synthetic
Intercept		0.20 (0.31)	−1.47 (0.31)***	−1.93 (0.36)***	−0.51 (0.2)***	−0.83 (0.22)***
Female (Ref: Male)		−0.40 (0.18)**	−0.40 (0.18)**	−0.33 (0.16)**	−0.46 (0.12)***	−0.03 (0.12)
Age	linear	−0.05 (0.22)	0.41 (0.25)	−0.18 (0.23)***	0 (0.16)	−0.08 (0.15)
	quadratic	−0.24 (0.19)	0.01 (0.22)	−0.55 (0.19)	−0.35 (0.14)**	−0.39 (0.13)***
	cubic	0.23 (0.15)	0.03 (0.16)	0.03 (0.14)	0.03 (0.11)	0.18 (0.10)*
Income	linear	0.54 (0.28)*	0.82 (0.30)**	NA	0.87 (0.20)***	0.45 (0.18)**
	quadratic	−0.09 (0.25)	−0.03 (0.27)	NA	−0.22 (0.17)	−0.09 (0.16)
	cubic	0.42 (0.24)*	0.48 (0.26)*	NA	0.24 (0.16)	0.18 (0.15)
Have a family member who uses bile (ref. does not)		2.24 (0.18)***	0.69 (0.23)***	1.88 (0.26)***	1.29 (0.13)***	1.06 (0.12)***
Have a friend or colleague who uses bile (ref. does not)		0.92 (0.2)***	−0.29 (0.20)	−0.24 (0.17)	0.58 (0.12)***	0.20 (0.12)*
Passed knowledge test by only selecting true treatment uses for bile (ref: did not pass)		−0.17 (0.18)	−0.81 (0.18)***	−0.81 (0.16)***	−0.33 (0.12)***	−0.24 (0.12)**
Thinks wild bile is illegal (ref: thinks it is legal or partly legal)		0.05 (0.19)	−0.64 (0.18)***	−0.70 (0.16)***	0.08 (0.12)	0.11 (0.12)
Thinks wild bears are decreasing in China (ref: does not think they are decreasing)		0.38 (0.21)*	−0.33 (0.22)	−0.26 (0.20)	0.13 (0.16)	0.29 (0.15)*
Passive in decisions about mild disease (ref: active)		0.16 (0.09)*	0.04 (0.09)	−0.13 (0.08)	0.05 (0.06)	0.07 (0.06)
Passive in decisions about severe disease (ref: active)		−0.03 (0.08)	−0.01 (0.08)	−0.11 (0.07)	0.04 (0.05)	−0.04 (0.05)
Lives in province with bear farms (ref: no farms)		0.13 (0.17)	0.02 (0.18)	−0.10 (0.17)	0.07 (0.12)	−0.01 (0.11)

^a^ Full model outputs in Appendix S9.

^b^ Significance: * = 0.05> *p* <0.1; ** = 0.01> *p* <0.05; *** *p* <0.01.

### Discrete choice experiment

We had 12,789 completed choice sets from 1421 respondents. The opt‐out was chosen in 9.3% of choice sets, with the most frequently selected opt‐out alternative being a different bear bile product (41.3% opt‐outs), followed by herbal alternatives (30.6%). A different animal bile was chosen in 4.8% of opt‐outs, with respondents specifying cow or snake bile because of lower prices.

**FIGURE 2:**
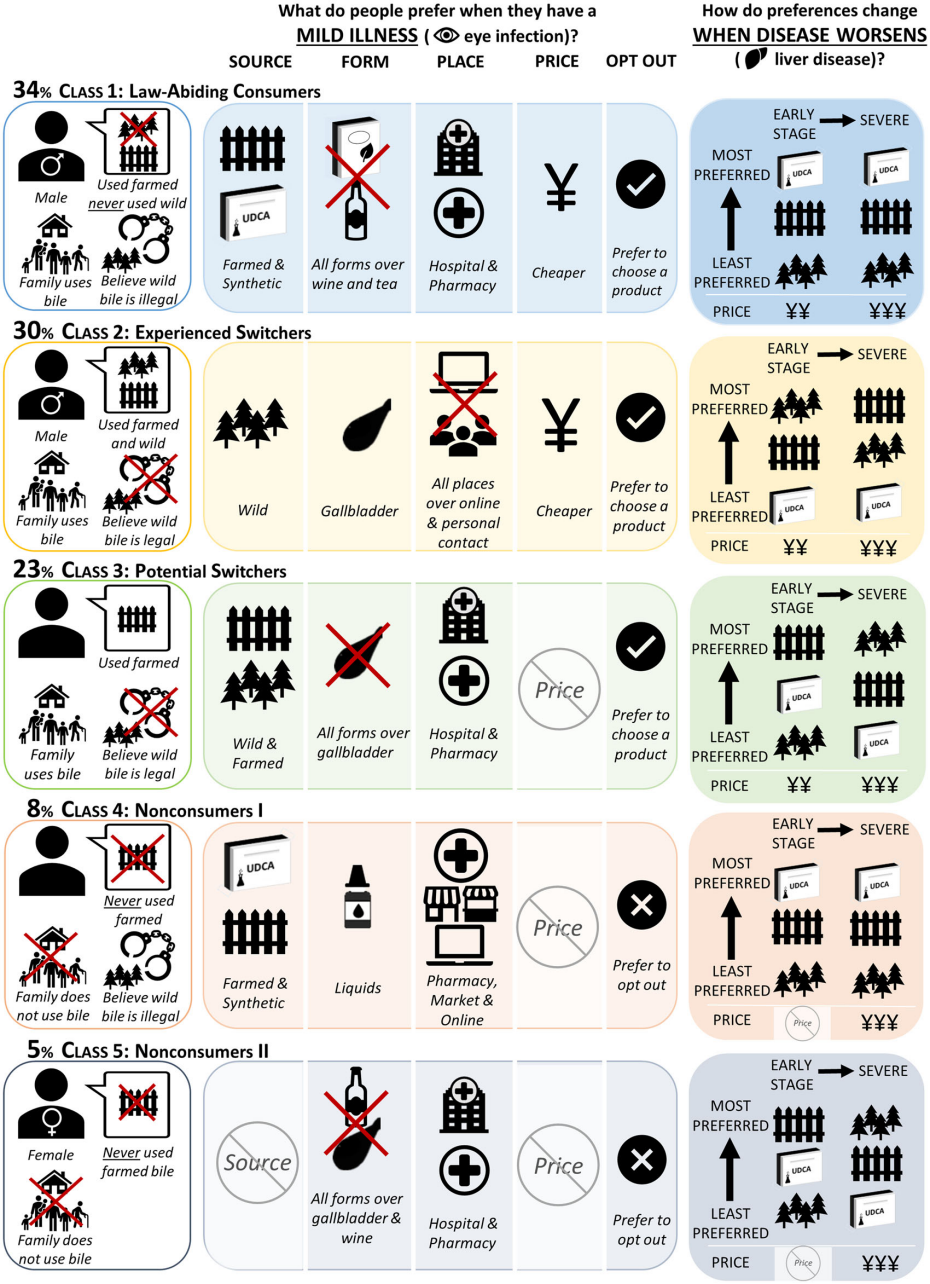
Summary of latent class model results identified using a discrete choice experiment on bear bile consumer preferences, showing the characteristics of class members and their preferences for bear bile products

In the LCMs, we selected a final model with five latent classes (Appendix S11). Shared preferences differed among classes across all attributes, and all class‐specific preferences changed as disease scenario worsened. Class membership was significantly predicted by previous consumption of farmed bile, previous consumption of wild bile, gender, knowing that wild bile was illegal, and having family members who used bile (Figure 2; Appendices S12 & S13).

We named each class based on our interpretation of broad class characteristics and preferences. Class 1 (34% of sample) was named *law‐abiding consumers* because they were more likely to know that wild bile is illegal and had never used it. They also showed marginal preferences for legal farmed and synthetic bile, with no increase in marginal preferences for wild bile even as disease worsens. This class showed the lowest propensity to switch to wild products because they preferred only legal products and formal vendors, although they preferred all product forms (including gallbladders) over wine and tea products. We combined classes 2 (30%) and 3 (23%) into one group and named them *all‐natural consumers* (53%) because they were more likely to have consumed nonsynthetic bile in the past and to dislike synthetic bile. They were also more likely to have family members who used bile and not know that wild bile is illegal. Class 2 was named *experienced switchers* because they were more likely to have consumed both wild and farmed bile in the past (and, therefore, must have switched at least once). They were more likely to prefer wild bile, gallbladders, and diverse places of purchase, making them the most likely class to purchase wild bile in the mild disease scenario, but they showed marginal preferences for farmed bile as disease worsens. Class 3 was named *potential switchers* because they were less likely to have used wild bile in the past. Their preferences for farmed bile and formal vendors combined with a dislike for gallbladders made them unlikely to seek wild bile out in mild disease scenarios. However, this changed as disease worsened, due to marginal preferences for wild bile in severe disease scenarios. Finally, classes 4 (8%) and 5 (5%) were less likely to have consumed bile in the past and knew no consumers in their family. Although their specific preferences for different attributes differed, both classes preferred to opt out rather than choose one of the bile products presented. We, therefore, combined them into a group named *nonconsumers*.

As disease scenarios progressed from mild to severe, all classes showed marginal preferences for more expensive products. Marginal preferences for farmed bile relative to wild strengthened in the early disease scenario for the law‐abiding consumers (class 1), potential switchers (class 3), and one nonconsumer group (class 5) and in the severe disease scenario for the experienced switchers. In addition, while marginal preferences for synthetic relative to wild strengthened in the early disease scenario for potential switchers, they weakened in both early and severe scenarios for the law‐abiding consumers (class 1).

### Willingness to pay

We could only calculate WTP for the two LCM classes for which the mean effect of price on choice was significant (law‐abiding consumers [class 1] and experienced switchers [class 2]) (Appendix S14). In the LCM, the law‐abiding consumers had the highest WTP for synthetic bile over wild (CNY4430 [US$645]) and buying in hospitals over pharmacies (CNY1215 [US$177]). Their WTP for liquids, powder, and tablets or capsules was not significantly different from gallbladders, but they would pay significantly less for wine (CNY1490 [US$217]) or tea (CNY939 [US$137]) compared with gallbladders. In contrast, experienced switchers would pay CNY903 (US$131) more for wild bile over farmed and CNY1020 (US$149) for wild over synthetic bile. Their highest WTP was for gallbladders over all other product forms (CNY761‐1262 [US$111‐184]). Although their WTP for buying from pharmacies, hospitals, and markets was not significantly different, their WTP for buying bile online was CNY1482 (US$216) less than at a pharmacy.

## DISCUSSION

**FIGURE 3:**
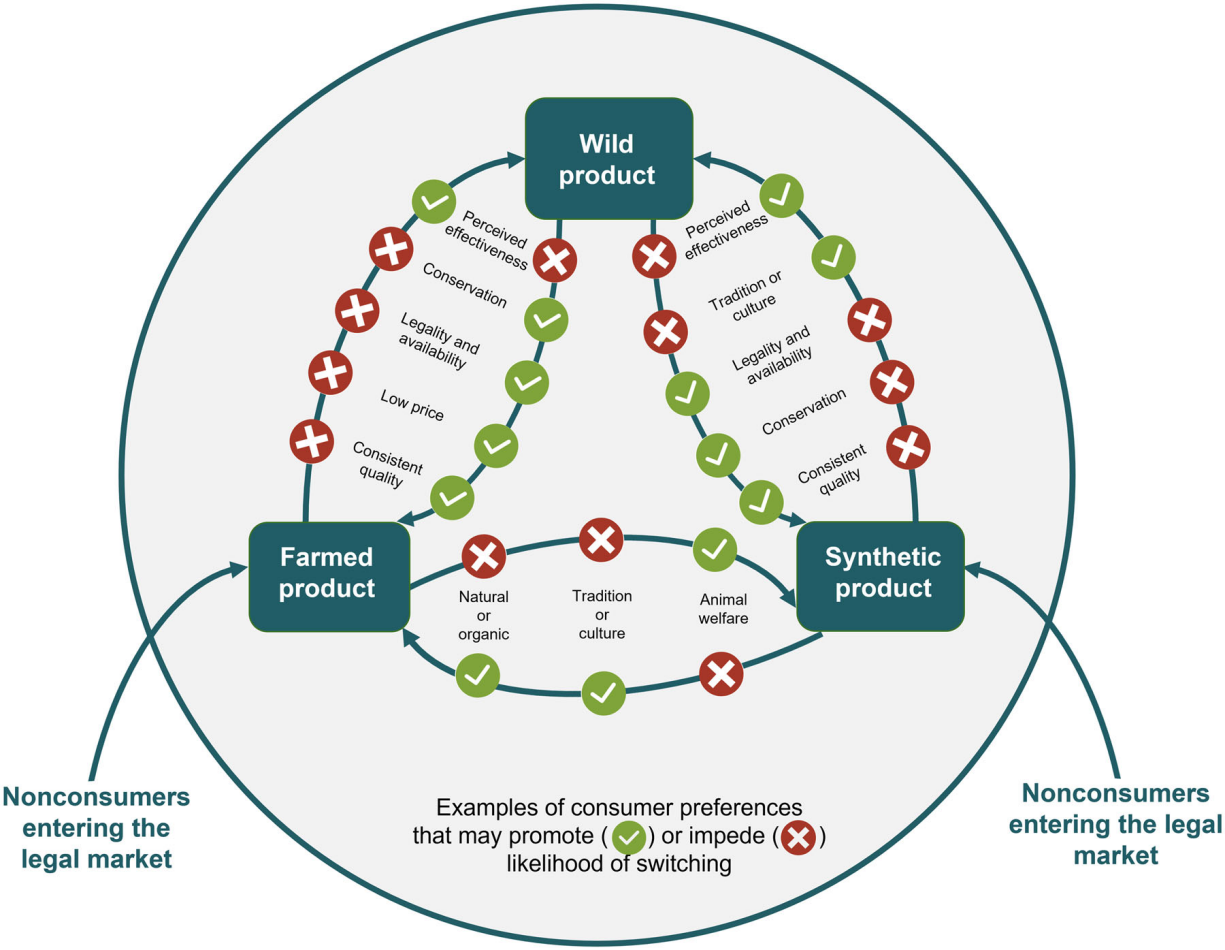
Examples of how individual consumer preferences may promote or impede the likelihood of switching between wild, farmed, or synthetic wildlife products. Specific preferences will be dependent on market context, consumer characteristics, and motivation to make a switch. Motivations for switching or entering the market may include change in personal wealth, change in availability or legality of a product, introduction of a new product to the market, or increased urgency of need for the product (e.g., illness)

We found that different wildlife consumers had distinct preferences for wild, farmed, and synthetic products and that these preferences influenced their likelihood of switching in either direction between illegal wild products and different legal alternatives (Figure 3). For the first time relative to these complex medicinal markets, we showed that the likelihood of consumer switching is affected by combinations of their knowledge and experiences (e.g., personal or family consumption), immediate motivation for consumption (e.g., disease severity), and preferences for product attributes beyond simply the product's source (e.g., place of purchase). The interplay between these factors will make certain consumers more likely to seek out wild products, whereas others will be less likely to do so if legal alternatives that match their preferences exist. Complex choices in wildlife markets have been demonstrated before (Liu et al., 2016; Hanley et al., 2017), as have specific examples of consumer uptake of nonwild alternatives (e.g., Naude et al., 2020), but our findings revealed how self‐reported consumers dealt with this complexity when making purchasing decisions. Our results provide an empirical example of the importance of integrating context‐specific understanding of distinct consumer behaviors into the design and evaluation of conservation interventions that aim to reduce illegal wildlife consumption, supporting calls by previous authors for this type of research (Hinsley & ‘t Sas‐Rolfes, 2020; Thomas‐Walters et al., 2020).

Although stated preference studies cannot record real switching behavior, our findings confirmed that self‐reported bear bile consumers have preferences that would underpin switching between products. Furthermore, the nature and likelihood of switching is likely to depend partly on whether people have consumed wild or farmed products in the past. This has implications for our current understanding of these markets because recommendations on the use of legal alternatives to reduce demand for wild products are often based on data from nonconsumers (e.g., Dutton et al., 2011). For example, stated preferences for synthetics often lead to recommendations that they could reduce demand for wild products (Davis et al., 2016; Liu et al., 2015, 2016), but this would require consumers of wild products to be willing to switch to these alternatives. In our sample, the only strong preferences for synthetic bear bile were among law‐abiding (class 1) and some nonconsumers, who both showed no past consumption of, and no preferences for, wild products. Conversely, all‐natural consumers, who were most likely to be wild‐bile consumers, strongly disliked synthetic products, suggesting that switches between farmed and synthetic bear bile would be more likely than direct switches from wild to synthetic. Although it is the only one with proven effectiveness, UDCA is just one component of bear bile, so our results may not be generalizable to markets where synthetics more closely mimic the form and composition of wild products (e.g., rhinoceros horn [Mi et al., 2019]). Promoting synthetics rather than farming for wildlife products may seem a beneficial option for both conservation and animal welfare, but there is a risk that some consumers will not be swayed without carefully targeted promotions, developed based on an understanding of their preferences (Doughty et al., 2021). Although our consumer groups will not directly map onto other markets, it is likely that past consumption experience will influence product preferences and willingness to try different alternatives in the future in many wildlife consumption contexts.

Our findings suggest that consumption experience and knowledge of a product may also influence how consumers change their behavior in response to changing external drivers of consumption, such as increasing disease severity. Many consumers in our study switched to different products as disease worsened, although these switches were not in a uniform direction. All consumers in our sample would pay more in these severe disease scenarios, suggesting that they were originally compromising on preferred attributes to save money when the need for a product is less urgent (Dutton et al., 2011). In addition, our CPS showed that consumers were reluctant to make their own treatment decisions when they were severely ill, supporting previous findings that medical practitioners are important sources of external advice on bear bile treatments in China (Hinsley et al., 2021). This could be related to consumers’ poor knowledge of how bile is used in treatment because when knowledge of a product is low, people employ heuristics based on social influence to make purchasing decisions (Wood & Hayes, 2012). The link between consumption of bile and having family members who also consume bile is consistent with other studies that showed that, in addition to doctors, informal advice from nonmedical sources is important (Dang Vu et al., 2020; Davis et al., 2019, 2020). Further work on traditional medicine markets could investigate family influences in more depth, including better understanding explicit versus implicit product preferences. Implicit preferences based on past experiences of consumption are particularly strong for traditional food products (Kimura et al., 2010). Our findings provide further evidence for the importance of considering external drivers of wildlife consumption when designing strategies to reduce it (Dang Vu et al., 2020) and highlight the importance of considering the heterogeneity of these drivers and their influence on consumer behavior.

Although past experiences and external drivers may influence which wildlife products are preferred, preferences for product attributes other than source are likely to mediate demand for illegal products. The full range of factors affecting wildlife consumer decisions are rarely considered and are likely to include legality, price, or social legitimacy (Davis et al., 2020; Hinsley & ‘t Sas‐Rolfes, 2020; Thomas‐Walters et al., 2020). We found that legality is likely to be a key factor in consumption and preference for wild bile; this association was found in classes that were also more likely to believe that wild bile was legal (the all‐natural consumers). Whether this reflects true beliefs or an attempt to justify consumption of these products is unclear, but poor knowledge of legality matches findings in other TCM markets (Wang et al., 2020). However, even where consumers believe products are legal, they may be dissuaded from purchasing illegal products by high prices, poor availability, or need to purchase from hard‐to‐find informal vendors. In China, illegal bear bile is often sold as an expensive raw product (CNY10,000 per wild gallbladder: Liu et al., 2011) and is unlikely to be sold in trusted places, such as hospitals, because of high penalties for medical practitioners engaging in illegal trade (Cheung et al., 2018). Therefore, even though our potential switchers preferred both farmed and wild products, they might never use wild products given their strong preferences for purchasing in hospitals and pharmacies. Similarly, while overall preferences for gallbladders, which are illegal, were found in our sample, consumers showed relatively low WTP for them over cheaper, widely sold, legal products, such as capsules.

Future efforts to understand illegal wildlife consumption must account for the complex interactions of different preferences when drawing conclusions about demand for wild products. They should also carefully consider whether the presence of legal alternatives normalizes the consumption of illegal products (Rizzolo, 2021) and how this effect can be reduced. Although education about legality has been recommended to reduce illegal wildlife consumption in China (Liu et al., 2015; Wang et al., 2020), this may not reduce rule breaking, especially when perceived risk of prosecution is low (Hanley et al., 2017). More nuanced messages, based on normative approaches with an emphasis on high levels of enforcement, may be more effective (Kahler & Gore, 2012), especially when combined with efforts to ensure that legal alternatives are priced competitively and that formal vendors are prevented from engaging in the illegal trade.

There are limitations to our study that must be considered when interpreting our results and planning future work. First, a revealed preference study would be the gold‐standard for understanding consumer preferences, but we chose to use the next best option of a nonhypothetical stated preference method (Hinsley & ‘t Sas‐Rolfes, 2020) because collecting real legal or illegal sales and price data in the Chinese bear bile market was not feasible. Although we went to great lengths to reduce hypothetical and social desirability biases, stated preference studies are still subject to these. Ideally, future work could try to develop methods for revealed preference studies of wildlife markets.

Although our DCE presented respondents with choices of all bile sources in each disease scenario, from a design perspective including these interactions as part of the DCE design could have reduced the potential for inefficient estimates. Some effects of disease worsening may also be due to learning effects. Further, despite their relative anonymity and ability to sample hard‐to‐reach groups, online samples often lead to overrepresentation of certain demographic groups (e.g., younger and more educated people). Our sample was, therefore, not representative of the national average (Appendix S7), especially because China is a large and diverse country.

We could not extrapolate relative class sizes or specific values (e.g., WTP figures) beyond our sample. However, while bear bile consumers are heterogenous, the preferences we found likely represented significant consumer groups within the Chinese market. Themes, such as reluctance to use synthetic versions of traditionally naturally derived products (e.g., food and medicines), may also apply to other wildlife markets.

Finally, we focused on preferences for products within ailment scenarios that would require doctor consultation or treatment with a medicinal product. However, wild or farmed bear bile is also used for other purposes, for example, as a health tonic or to reduce heatiness, which might not always require formal medical intervention (Hinsley et al., 2021). Future studies could investigate in more detail these milder conditions that may drive bear bile use, especially as findings in neighboring countries show that bile is used extensively for mild conditions, such as bruising (Davis et al., 2019).

Our findings add to a growing literature demonstrating the complexity of wildlife markets, with different consumers switching in both directions between legal and illegal products. These behaviors depend on an array of factors, including price, availability, legality, and severity of disease, as well as the accuracy of consumer knowledge of product and market attributes. In line with the concept of medical pluralism, we emphasize the need to consider that the same consumer may switch between different bear bile or alternative products multiple times or may use different products concurrently. It is, therefore, difficult to answer conclusively the fundamental question about whether farmed products, such as bear bile, encourage more people to switch away from wild products or add new consumers who later switch to (or at least occasionally try) wild products. However, our findings suggest that under current market conditions in China, consumers like those in our sample may be unlikely to seek out wild bile unless it becomes more accessible to them or their preferred alternative is unavailable.

We suggest that supply‐side approaches to provide legal alternatives to consumers should account for complexity of switching behavior. Promoting different legal alternatives to appeal to different consumers and ensuring variations in price, form, and other attributes is key to better compete with wild (often illegal) products. The most appropriate substitutes will vary between markets but may include those from different species (e.g., herbal alternatives to animal‐based products [Moorhouse et al., 2020]), as well as synthetics of different types and farmed or sustainably wild‐sourced legal alternatives. This approach, combined with better law enforcement to prevent illegal products from being sold in trusted and easily accessible places (e.g., pharmacies), may help inflate the cost of buying wild‐sourced products. To encourage consumers to switch, intervention messaging should be framed around the attributes of legal products most preferred by the consumers of wild products, rather than the broader public. In addition, where applicable, the high likelihood of being caught and punished for consuming illegal products could be highlighted. Ideally, interventions could focus on consumers and key influencers, such as family members, who may sway purchasing decisions (Doughty et al., 2021). Finally, we recommend that in‐depth work with actual consumers must be prioritized when trying to understand and predict changes in illegal wildlife markets and that the complexity of consumer behavior should be central to all interventions that aim to induce these changes.

## Supporting information

Supporting Information
