# Supplementary material for: Understanding why consumers in China switch between wild, farmed, and synthetic bear bile products
Source: Conserv Biol. 2022 Apr 29;36(3):e13895. doi: 10.1111/cobi.13895 (PMC9320993; doi:10.1111/cobi.13895)
Supplement: Supplementary file 1 — Supporting Information [file COBI-36-0-s001.docx]

**Supplementary Materials**

***S1*** *Sample filtering process, quality control and consistency checks…………****p2***

***S2.*** *Pilot surveys…………………………………………………..……………….****p4***

***S3****. Attribute combinations used in the DCE…………………………..………..****p5***

***S4****. Main consumer survey questions and answer options ………………….****p6***

***S5.*** *Covariates used in models………………………………………………….****p12***

***S6.*** *Utility…………………………………………………………………………..****p14***

***S7.*** *Sample characteristics………………………………………….…………..****p15***

***S8.*** *Bile use in our sample………………………………………….………...…****p17***

***S9.*** *Full model averaging results for general linear models for different bear bile consumer types…………………………….…………...****p18***

***S10.*** *The Random Parameter Logit model for the DCE……….………….…****p35***

***S11.*** *Latent class estimation and model selection…………….……............****p36***

***S12*** *Descriptive statistics of respondents in each LCM class.………..…...****p37***

***S13.*** *Results of the 5-class LC model for the DCE….………….…………..****p38***

***S14.*** *Willingness to Pay estimates for both Latent Class Model classes with significant price attributes………….……………****p39***

*References for all Appendices……………………………………….……....****p40***

***S1. Sample filtering process, quality control and consistency checks***

Online samples can be at risk from automated or non-automated ‘fake’ answers if controls are not put on the means of accessing the survey, and if the quality of responses is not checked (Bybee et al. 2021). Our final sample was therefore obtained via the following means:

*Sample filtering*

Wen Juan Xing (WJX) randomly invited members of its panel and then presented them with pre-survey questions regarding TCM usage, awareness of bear bile, age and place of residence. We included only respondents who reported that they had personally bought and/or used TCM products in any form (including ingredients, processed products, or treatments), either by prescription or over the counter and who had heard of bear bile. Additionally, only those aged 18+ who were resident in mainland China could progress to the survey, where they saw a consent script and had to confirm they would like to proceed.

*Quality control (QC)*

WJX’s post-survey quality control (QC) process cut out 733 respondents, leaving a sample of 1110. Respondents were cut if they: took more or less time than the standard 3-5 seconds per question; gave inconsistent responses; consistently selected the same answer (e.g. the first option for every multiple-choice question); failed trivia questions inserted into the survey; or gave erroneous answers (e.g. random letters in open text questions). However, during analysis it became clear that large numbers of respondents that had failed the QC process were not in violation of these criteria. For example, several people who had been cut out had given detailed and highly-relevant answers to open text questions throughout the survey. In addition, potentially due to the ‘inconsistent answers’ criteria, there was some bias in the respondents who were cut, including more than half of those who stated they had used wild bear bile or a bear gallbladder. Due to concerns with the applicability of these criteria to our particular survey, we developed our own QC process using the following protocol:

- **Stage 1:** Were open text box comments coherent, showing understanding of the question and survey. **Yes** = pass QC; **Unsure** (e.g. generic response such as ‘I liked the survey’ = Progress to Stage 2; **No** (e.g. nonsense responses) = Fail QC.
- **Stage 2:** Were there clear patterns in answers that cannot be explained by survey design (e.g. all choice experiment answers were ‘A’). **Yes** = Pass. **No** = Stage 3.
- **Stage 3.** For remaining surveys, was the completion time in the top or bottom 5% of times in the sample (controlling for those who answered more/fewer questions due to survey logic). **Yes** = Fail. **No** = Pass.

*Consistency checks*

In addition, for the discrete choice experiment, we wanted to understand how consistent respondents were at the beginning and end of the experiment, so we included a transitivity test. This was a set of three 2x2 choice sets presented at the end of the 9 choice sets, replicating a choice set already seen by the respondent. It was presented as a set of binary choices (e.g. original choice set would be one question presenting ‘A or B or C’ and transitivity test would be three questions presenting ‘A or B’, ‘B or C’, ‘A or C’). In theory, if choices are consistent, a respondent who chooses A in the original choice set should choose A over B and A over C when presented with those same choice cards again, even in a different format. In our final sample, we found that 62% of respondents showed complete consistency with their original answer, increasing to 91% of respondents when those who showed partial consistency were included.

***S2. Pilot surveys***

Pilot 1: to gather qualitative data on attributes, levels and experiment framing

*March 2018, face-to-face with 32 members of the public in Guangzhou*

In addition to the structured CE questions, each respondent was asked to explain their choices, discuss any issues they faced in answering the questions, and give feedback about the survey. These qualitative data combined with the results of the CE were used to clarify the descriptions of attributes and levels, the levels included, and improve the instructions given to respondents completing the survey. In particular, this pilot phase revealed that people were confused about the reason they would be taking bile, so we added a medical condition scenario to each choice card. These were chosen based on the most commonly cited uses of bear bile in a survey of the public in four Chinese provinces carried out by the authors: heatiness, eye problems and liver disease.

Pilot 2: to provide priors to produce a D-efficient Bayesian design.

*February 2019, 162 respondents (102 non-consumers and 59 consumers) online via WJX*

Following this pilot, we changed the medical condition scenarios to remove the very mild condition ‘heatiness’, as respondent feedback suggested that people found it hard to justify choosing any formal treatment for this condition, instead suggesting that they would just rest. Instead we split the liver disease condition into mild and severe, to better understand how severity of condition affects choice. We also added clearer instructions for respondents on how to complete the choice questions.

Testing phase

*June-July 2019*

To test the final experiment with randomly selected respondents from the panel, for understanding and to flag any potential technical issues. Midway through the testing phase one addition was made to one of the behaviour questions (We added ‘would you ever use synthetic bile in the future?’) but no changes to the core questions were made and no issues related to understanding of questions or technical issues were flagged, so the pilot results were used in the final sample.

***S3. Attribute combinations used in the DCE***

Attribute combinations used in the DCE. All feasible possibilities, including combinations with relatively small chances of occurrence, were included (e.g. wild bile is rarely sold in pharmacies, but has been observed by the authors at least once, while synthetic UDCA cannot be sold as a gallbladder, so that option was not included).

| Source of bile | Place of purchase | Product form | Price per course of treatment (CNY) |
| --- | --- | --- | --- |
| Wild | Online; Personal contact; Pharmacy; TCM market | Gallbladder; Powder; Medicinal wine | 1000; 2500; 5000 |
| Farmed | Farm; Hospital; Online; Personal contact; Pharmacy; TCM market | Powder; Medicinal wine; Liquids (inc. eyedrops); Tablets/Capsules; Tea | 25; 250; 500; 1000; 2500 |
| Synthetic | Hospital; Online; Personal contact; Pharmacy; TCM market | Liquids (incl. eyedrops); Tablets/Capsules | 250; 500 |

***S4. Main consumer survey questions and answer options***

Complete survey in English, excluding support icons and codes for question branching, requirements, randomization and page settings. Notes indicated in blue italics, with direct English-to-Chinese translation references.

| **Survey on consumer attitudes towards bear bile products** | | | | | |
| --- | --- | --- | --- | --- | --- |
| **Eligibility I** | 1 | Are you over the age of 18? | Yes/No *<INVALID>* |  |  |
|  | 2 | Do you work and live in mainland China? | Yes/No *<INVALID>* |  |  |
| **Survey background** | - | This survey is part of a project focussed on attitudes to Traditional Chinese Medicine being carried out by <removed for blinding purposes during review>. If you take part, it will take 15 minutes and you will not have to give your name or any identifying information. This survey may include questions that could be perceived as sensitive, but no respondents will be identified for this study, and thus there is no chance for your answers to be linked in any way back to you. The overall results of this project may be published, but you don’t have to take part if you do not want to, you can withdraw at any stage without giving a reason and without any negative consequences.  Upon completion, you will be rewarded with WJX member points worth <RMB10.  The University of <removed for blinding purposes during review> are the data managers, but the data may be shared with other project partners and related organizations, used in future studies, and shared with other researchers (e.g., in online databases). The data that we collect from you may be transferred to, and stored or processed at, a destination outside your country. By submitting your information, you agree to this transfer, storing or processing.  This project has been reviewed by, and received ethics clearance through, the <removed for blinding purposes during review> university ethics committee. If you have a concern about any aspect of this project, please speak to the researcher, who will do their best to answer your query, or please contact <removed for blinding purposes during review>. with the reference number R54657/RE002. | | |  |
| **Eligibility II** | 3 | Have you seen or heard of bear bile products? | Yes/No *<INVALID>* |  |  |
| **Consent** | 4 | Do you agree to take part? | Yes/No *<INVALID>* |  |  |
| **Demographics I** | 5 | Which province do you currently reside in? | *<Drop down list of all provinces and municipalities in China>* |  |  |
|  | 6 | Which province is your hometown in? | *<Drop down list of all provinces and municipalities in China>* |  |  |
| **Medical preferences & habits** | 7 | When was the last time you visited any kind of **Traditional Chinese medicine practitioner**? | Within 1 week  Within 1 month  Within 6 months  Within 1 year  1 year ago  [I have] never been  [I have] forgotten |  |  |
|  | 8 | When was the last time you visited any kind of **Western medicine practitioner**? |  |  |  |
| **Control Preference Scale** | 9 | Imagine if you have a **mild illness (such as an eye infection)**. Which of the following ways would (best) describe how you make decisions about which treatment you will use? | *<Active>* I prefer to make the decision about which treatment I will receive  *<Active>* I prefer to make the final decision about my treatment after seriously considering my doctor's opinion.  *<Collaborative>* I prefer that my doctor and I share responsibility for deciding which treatment is best for me  *<Passive>* I prefer that my doctor makes the final decision about which treatment will be used, but seriously consider my options.  *<Passive>* I prefer to leave all my decisions regarding my treatment to my doctor. |  |  |
|  | 10 | Imagine if you have a **serious illness (such as severe liver disease)**. Which of the following ways would (best) describe how you make decisions about which treatment you will use? |  |  |  |
| **Bile consumption** | 11 | When was the last time you bought bear bile products? | Within 1 week  Within 1 month  Within 6 months  Within 1 year  1 year ago  [I have] never bought before  I have forgotten |  |  |
|  | 12 | What types/forms of bear bile products have you **ever** bought/used ? Please select all that apply:  *Please note: In this survey, aqueous medications refer to aqueous solutions with specified medicinal properties, such as eye drops and oral liquids, and does not include medicinal wine and herbal teas.* | Liquids (Eyedrops only)  Liquids (Other)  Powder  Gallbladder  Cream  Tablets/Capsules  Pellets and granules  Medicinal wine  Tea  Other (Please specify)  I have never bought or used bear bile products |  |  |
|  | 13 | Please tell us more about the bear bile products you have used:  a) Products with farmed bear bile | Have often used [regularly]  Have not often used, used only once or twice)  Have never used  Not sure if I have used |  |  |
|  |  | b) Products with wild bear bile |  |  |  |
|  |  | c) Products with synthetic UDCA |  |  |  |
|  | 14 | What kinds of places have you bought bear bile products from? Please select all that apply. | TCM Market  Medical clinic  Hospital  Pharmacy  Bear bile farm  Online  Direct personal contact to seller  Received as a gift from others  Other (Please specify) |  |  |
|  | 15 | Have you bought/used bear bile products for any of the following reasons? | As medicine  To improve health condition  As a gift  Other (Please specify) |  |  |
|  | 16 | Would you consider using bear bile products for any of the following reasons in the future? |  |  |  |
|  | 17 | Do you personally know anybody else who has used bear bile products? | Father/mother  Grandfather/grandmother  Children  Husband/wife  Other family relatives  Friends  Colleagues  Other (Please specify)  I do not know |  |  |
| **Knowledge test on bile uses** | 18 | Do you know what bear bile products are used for? Please select any of the following options that you believe are true (Please select all that apply).  *<With time limit>* | *<TRUE>* Liver care  *<TRUE>* Eyesight  *<TRUE>* Detoxification and 'clearing heat'  *<FALSE>* Improve brain function  *<FALSE>*  Nourishing kidney  *<FALSE>* Increase calcium  No suitable option available |  |  |
| **Choice experiment: Guide** | - | For the next 12 questions you will be asked to read the descriptions of three products and use the information provided to decide which, if any of them, you would buy. Your answers to these questions will be anonymous and there is no way any of your answers can be traced back to you.  Please read the following so that you understand the types of products before you start:  (By selecting "I understand", you acknowledge that you have full understanding of the information provided). | | |  |
|  | 19 | Products containing farmed bear bile:  This product is made from bear bile that is extracted from bears that are raised in farms. | I understand |  |  |
|  | 20 | Products containing wild bear bile:  This product is obtained or made from bear bile that is taken from bears hunted in the wild. | I understand |  |  |
|  | 21 | Products containing synthetic UDCA:  This product contains only the active ingredient from bear bile and is synthesised in a laboratory, not taken from a bear. | I understand |  |  |
| **Choice experiment: Consent** | - | The survey questions will be displayed in the format shown in the following image, with the specifications of each product described in text and information aligned for comparison. Considering the given hypothetical medical situation, when making your decision, you can choose to purchase one of the listed products with the most preferred specifications for treatment, or not to purchase any of the above. | | |  |
|  | 22 | Do you promise that you will answer the following questions honestly, as if this was a real life situation?  *Please note: As the context of the questions may vary slightly, please read carefully before you fill in your answers.* | Yes, I promise  No, I cannot promise that |  |  |
| **Choice experiment**  *<Block A/B >* | 23-25 | Imagine that you have visited your doctor and they have told you that you have an **eye infection**. They have suggested that bear bile products could be an appropriate treatment. They have not told you what type of bear bile products to buy or where to get it from. There are multiple product options available in the market for purchase. Which of the following products would you use? | Purchase Product A  Purchase Product B  Purchase Product C  I would not purchase any of the above |  |  |
|  | 26-28 | Imagine that you have visited your doctor and they have told you that you have **mild liver disease**. They have suggested that bear bile products could be an appropriate treatment. They have not told you what type of bear bile products to buy or where to get it from. There are multiple product options available in the market for purchase. Which of the following products would you use? | Purchase Product A  Purchase Product B  Purchase Product C  I would not purchase any of the above |  |  |
|  | 29-31 | Imagine that you have visited your doctor and they have told you that you have **severe liver disease**. They have suggested that bear bile products could be an appropriate treatment. They have not told you what type of bear bile products to buy or where to get it from. There are multiple product options available in the market for purchase. Which of the following products would you use? | Purchase Product A  Purchase Product B  Purchase Product C  I would not purchase any of the above |  |  |
|  | - | *<Follow-up after selecting Q23-31 “I would not purchase any of the above”>*  a) Apart from the options listed, what would you choose to do instead? | Ask for another bear bile product  Ask for an animal bile alternative (please specify)  Ask for a herbal alternative (please specify)  I will do nothing  I do not know |  |  |
|  | 32-34 | *<Transitivity questions for evaluation. Based on Q23, with the choice set split into three versions, all of which had two product choices instead>* | Purchase Product A  Purchase Product B  I would not purchase any of the above |  |  |
| **Sequential ranking**  *<best choice*  *preferences>* | 32 | Imagine if you have been diagnosed with **mild liver disease** and are currently in discomfort. The following drugs are suitable treatments for your condition. Please select your most preferred and perceived most effective choice of treatment. | *<TCM-Bear>* Products containing farmed bear bile  *<TCM-Bear>* Products containing wild bear bile  *<TCM-Synthetic>* Products containing synthetic UDCA  *<TCM-Animal alternative>* Products containing farmed snake bile  *<TCM-Animal alternative>* Products containing wild snake bile  *<TCM-Herbal alternative>* Products containing farmed huanglian  *<TCM-Herbal alternative>* Products containing wild huanglian  *<TCM>* [I would seek] Other traditional Chinese medicine/alternative treatments (Please specify)  *<Western medicine>* (I would seek) Other western medicine/alternative treatments (please specify)  *[Doctor’s decision]* I would let my doctor have full authority to make the decision, and [I] will not intervene or include any additional input myself (Please indicate your reference to a TCM/Western doctor) |  |  |
|  | 33 | Imagine that some time has passed, and you have now been diagnosed with **severe and potentially life-threatening liver disease**. In this situation (your condition), which of the following treatments would you consider to try? |  |  |  |
|  | 34 | Severe and potentially life-threatening liver disease: Imagine that you have used your previous choice of treatment, and it has been **less effective** [to your condition] than hoped. In this situation, which of the following treatments would you consider to **try next** (or would you continue to use the same [form of treatment])? |  |  |  |
|  | 35-38 | Imagine that you have used your previous choice of treatment, and it has **still been less effective** [to your condition] than hoped. In this situation, which of the following treatments would you consider to try next (or would you continue to use the same [form of treatment])? |  |  |  |
| **Likert scale** *<Behaviour intent>* | 34 | How likely are you to buy products containing farmed bear bile in the future? | Very likely  Somewhat likely  Neither likely nor unlikely  Somewhat unlikely  Very unlikely |  |  |
|  | 35 | How likely are you to buy products containing wild bear bile in the future ? |  |  |  |
|  | 36 | How likely are you to buy products containing wild bear bile in the future ? |  |  |  |
| **Knowledge II** | 37 | Currently, wild bear populations in China are: | Increasing  Decreasing  Stable [no change]  I do not know |  |  |
|  | 38 | Currently, buying wild bear bile in China is: | Legal  Illegal  [There are] both legal and illegal  I do not know |  |  |
| **Demographics II** | 39 | Please state your gender: | Male  Female  Other  Prefer not to say |  |  |
|  | 40 | Please state your age: | 18-24  25-34  35-44  45-54  55-64  65-74  75-84  85+ |  |  |
|  | 41 | What is your current employment status? | Full time  Part time  Housewife/husband  Student  Unemployed  Retired  Self-employed  Other (please specify) |  |  |
|  | 42 | If [you are] full-time employed, what is your profession? | *<Drop down list of main industries/professions >* |  |  |
|  | 43 | What is your annual income? Please select the category that applies to you best. | Less than CNY 10,000  CNY 10,000 - 50,000  CNY 50,000 - 100,000  CNY 100,000 - 150,000  CNY 150,000 - 200,000  More than CNY 200,000 |  |  |
|  | 44 | What is your highest level of education? | None  Elementary school  Middle School  High school  University undergraduate  Masters  Doctorate |  |  |
| **Survey evaluation** | 45 | Many thanks for taking part in this survey.  What are your thoughts about this survey? Any feedback or comments are welcome. | *<Optional open text>* |  |  |
|  | - | This work is part of a larger project looking at the use of bear bile and its alternatives by people in China, specifically how many people use these products and why people choose to do so. None of the answers can be tracked back to you. In case of any concerns or complaints, the project’s ethics reference number is R54657/RE002 and you can contact <removed for blinding purposes during review>. who will deal with your concerns.  Thank you for your time. | | |  |

***S5. Covariates used in models***

| **Variable** | **Levels used in analysis** | **Description** | **Justification for inclusion in models** |
| --- | --- | --- | --- |
| Resident of province which has bear farms (binary) | - Province with bear farms - Province with no bear farms | Based on question ‘Which province do you live in?’ combined with author data on bear farm locations. | Greater availability of farmed bile has been suggested to encourage consumption (Drury, 2009). We hypothesise that farmed bile would be more readily available in provinces with bear farms. |
| Age (ordinal factor) | - 18-24 - 25-34 - 35-44 - Over 45 | For models, the original levels of 45-54, 55-64, 65-74, 75-84, and 85+ combined into ‘Over 45’, due to smaller sample sizes in these groups. | Younger people consume more wildlife in Southern China (Zhang et al. 2008), while older people used more traditional medicine products in Beijing (Liu et al 2016). |
| Gender (binary) | - Male - Female | For models, the original category of ‘Other’ and ‘Prefer not to say’ were removed from analysis due to small sample size (n=10). | Men reported to be more likely to use animal-based TCM products and bear bile in other studies (e.g. Zhang et al. 2008; Zhang & Yin, 2014). |
| Annual income (ordinal factor) | - < 10,000 - 10,000 - 50,000 - 50,000 - 100,000 - 100,000 - 150,000 - 150,000 - 200,000 - > 200,000 | A six-level ordinal factor of annual income in Chinese yuan was used in all models. | Higher incomes have been linked to bile use in Vietnam (Drury, 2009). |
| Knowledge of TCM uses for bear bile (binary) | - Failed TCM use of bear bile knowledge test - Passed TCM knowledge test | A ‘pass’ was selecting bear bile as something that could be used to treat ‘real diseases (eye infection, liver diseases, heatiness) without selecting any ‘false’ uses (improving brain power, building calcium, treating kidney disease) | Users of animal-based TCM products have been found to have little knowledge about uses of different ingredients (Liu et al. 2016). |
| Control Preference Scale: Preferred role in treatment decisions for a mild disease (ordinal) | - Active - Neutral - Passive | Due to small sample size in the most passive level (*’I prefer to leave all my decisions regarding my treatment to my doctor’*) it was combined with the second most passive (’*I prefer that my doctor makes the final decision about which treatment will be used, but seriously consider my opinion’* ) for analysis. | TCM doctors at formal hospitals are unlikely to prescribe illegal products (Wang et al 2020). Severity of disease has been linked to willingness to use wild products (Dutton et al. 2011) |
| Control Preference Scale: Preferred role in treatment decisions for a severe disease (ordinal) | - Active - Neutral - Passive | Due to small sample size in the most active level it was combined with the second most active (’*I prefer to make the final decision about my treatment after seriously considering my doctor's opinion*’) for analysis. |  |
| Legality (binary) | - Do not think wild bile is illegal - Think it is illegal |  | Legality has been cited as an important factor influencing willingness to consume wildlife products (Dutton et al. 2011) |
| Status of wild bear populations (binary) | - Do not think wild bears are declining - Think that they are declining |  | Conservation awareness has been linked to willingness to use synthetic substitutes to wildlife products (Liu et al. 2016). |

***S6.* Utility**

Utility for bundle *i* can be expressed as a deterministic function $V_{i}$ and a stochastic error term $\epsilon_{ni}$. The indirect utility function for bundle *i* for individual *n* can be expressed as:

$V_{i}=ASC_{n}+\beta_{n}x_{i}$ Equation 1

*Random parameters model*

In a random parameters model, the probability that an individual *n* selects alternative *i* is expressed as:

$P_{\mathrm{ni}}=\frac{\exp\left( {x'}_{\mathrm{ni}}\beta_{n} \right)}{\sum_{j=1}^{J} \exp\left( {x'}_{\mathrm{nj}}\beta_{n} \right)}$ Equation 2

*Choice experiment: Latent Class Model*

Latent class models assume heterogeneity across respondents that varies across *S* classes. Certain observable characteristics (Z_n_) can be used to predict latent class membership using a multinomial logit model. The probability that an individual *n* in class *s* will select alternative *i* can be expressed as:

$P_{ni|s=\frac{\exp\left( {x'}_{\mathrm{ni}}\beta_{s} \right)}{\sum_{j=1}^{J} \exp\left( {x'}_{\mathrm{nj}}\beta_{s} \right)}}$ Equation 3

And the probability that an individual belongs in class *s* is expressed as:

$P_{\mathrm{ns}}=\frac{\exp\left( {z'}_{n}\delta_{s} \right)}{\sum_{s=1}^{S} \exp\left( {z'}_{n}\delta_{s} \right)}$ Equation 4

*Willingness to Pay*

The WTP for attribute *x* (e.g. product form) can be calculated as the ratio of the parameter on the attribute of interest ($\beta_{x}$) with the cost coefficient ($\beta_{price}$), shown in Equation 1.

$WTP_{x}=\frac{\beta_{x}}{\beta_{price}}$Equation 5

***S7. Sample characteristics***

| Variable | Level | Final sample  (n=1421) | | Chinese 2018 databook (where applicable) |
| --- | --- | --- | --- | --- |
|  |  | n | % sample | % population |
| Live in a province with a bear farm* | Yes | 624 | 43.91 | 41.93 |
|  | No | 797 | 56.09 | 58.07 |
| Gender | Male | 559 | 39.34 | 51.17 |
|  | Female | 847 | 59.60 | 48.83 |
|  | Other | 1 | 0.07 | NA |
|  | Prefer not to say | 14 | 0.21 | NA |
| Age | 18-24 | 163 | 11.47 | 10.10 |
|  | 25-34 | 629 | 44.26 | 22.67 |
|  | 35-44 | 419 | 29.49 | 20.32 |
|  | 45-54 | 179 | 12.60 | 24.17 |
|  | 55-64 | 14 | 0.99 | 10.72 |
|  | 65-74 | 1 | 0.07 | 7.35 |
|  | 75-84 | 1 | 0.07 | 4.47 |
|  | 85+ | 0 | 0.00 | 1.10 |
| Education level | No school | 0 | 0.00 | 5.27 |
|  | Primary | 5 | 0.35 | 25.23 |
|  | Secondary | 59 | 4.15 | 55.61 |
|  | Undergraduate | 1146 | 80.64 | 13.27 |
|  | Masters | 189 | 13.30 | 0.60 |
|  | PhD | 22 | 1.55 |  |
| Income | < CNY 10,000 | 126 | 8.87 | Not recorded. But average income of employed people in urban areas was CNY 82,413 in 2018 |
|  | CNY 10,000 - 50,000 | 197 | 13.86 |  |
|  | CNY 50,000 - 100,000 | 370 | 26.04 |  |
|  | CNY 100,000 - 150,000 | 385 | 27.09 |  |
|  | CNY 150,000 - 200,000 | 207 | 14.57 |  |
|  | > CNY 200,000 | 136 | 9.57 |  |
| Employment | Full time | 1216 | 85.57 | Not recorded |
|  | Part time | 57 | 4.01 | Not recorded |
|  | House husband/wife | 19 | 1.34 | Not recorded |
|  | Student | 68 | 4.79 | Not recorded |
|  | Unemployed | 7 | 0.49 | 0.68 |
|  | Retired | 51 | 3.59 | Not recorded |
|  | Self-employed | 3 | 0.21 | 11.51 |
| Visits to medical practitioners in past year | Visited only TCM practitioners | 71 | 5.00 |  |
|  | Visited only ‘Western’ practitioners | 83 | 5.84 | Not recorded |
|  | Visited both ‘Western’ and TCM practitioners | 1207 | 84.94 | Not recorded |
|  | Visited neither | 26 | 1.82 | Not recorded |
|  | Forgotten/Not sure | 34 | 2.39 | Not recorded |

*According to NFGA statistics, in 2018 there were bear farms in Heilongjiang, Jilin, Liaoning, Sichuan, Yunnan, Zhejiang, Jiangxi, Guangdong, Guangxi, Shaanxi, and Fujian provinces. Both our results and official data book proportions derived from summing respondents/population in these provinces.

| **Category of bile use** | **Type of use** | **% of sample (n)** |
| --- | --- | --- |
| *Reason for purchase* | Medicine | *71.8% (1021)* |
|  | Health tonic | *51.8% (737)* |
|  | Gift | *14.9% (212)* |
| *Source of bile* | Wild | *16.7% (238)* |
|  | Farmed | *56.2% (799)* |
|  | Synthetic | *53.3% (758)* |
| *Form of bile used* | Powder | *20.0% (284)* |
|  | Other medical liquids | *12.7% (181)* |
|  | Eye-drops | *37.8% (537)* |
|  | Cream/Paste | *11.8% (167)* |
|  | Tablets/Capsules | *30.6% (435)* |
|  | Gallbladder | *18.2% (258)* |
|  | Tea | *1.1% (15)* |
|  | Granules | *16.1% (228)* |
|  | Wine | *18.2% (258)* |
|  | Other | *0.4% (6)* |
| *Place of purchase* | Pharmacy | *59.1% (841)* |
|  | Hospital | *53.8% (764)* |
|  | TCM Market | *27.1% (385)* |
|  | Clinic | *17.6% (250)* |
|  | Online | *16.1% (228)* |
|  | Personal Contact | *7.3% (103)* |
|  | Bear farm | *6.5% (93)* |
|  | Received as gift | *8.6% (122)* |
|  | Other | *0.4% (5)* |
|  | Never bought | *14.8% (210)* |

***S8. Bile use in our sample***

Summary of bile use reported by our sample (n=1421), focusing on the attributes of bile use that match our Discrete Choice Experiment.

***S9 Full model averaging results for general linear models on socio-demographic covariates between bear bile consumer types***

9.1 Full results of model-averaging for five generalised linear models using type of bile consumed as the dependent variable (n=1395). Shading denotes significance of at least p <0.05 (positive coefficients in yellow, negative in grey). Significance: * = 0.05> p <0.1 ** = 0.01> p <0.05, *** p <0.01

| Covariate | | Consumed any bile | Specific bile type consumed | | | |
| --- | --- | --- | --- | --- | --- | --- |
|  |  |  | Higher-certainty wild | Wild | Farmed | Synthetic |
| Intercept | | 0.20 (0.31) | -1.47 (0.31)*** | -1.93 (0.36)*** | -0.51 (0.2)*** | -0.83 (0.22)*** |
| Female (Ref: Male) | | -0.40 (0.18)** | -0.40 (0.18)** | -0.33 (0.16)** | -0.46 (0.12)*** | -0.03 (0.12) |
| Age | Linear | -0.05 (0.22) | 0.41 (0.25) | -0.18 (0.23)*** | 0 (0.16) | -0.08 (0.15) |
|  | Quadratic | -0.24 (0.19) | 0.01 (0.22) | -0.55 (0.19) | -0.35 (0.14)** | -0.39 (0.13)*** |
|  | Cubic | 0.23 (0.15) | 0.03 (0.16) | 0.03 (0.14) | 0.03 (0.11) | 0.18 (0.10)* |
| Income | Linear | 0.54 (0.28)* | 0.82 (0.30)** | NA | 0.87 (0.20)*** | 0.45 (0.18)** |
|  | Quadratic | -0.09 (0.25) | -0.03 (0.27) | NA | -0.22 (0.17) | -0.09 (0.16) |
|  | Cubic | 0.42 (0.24)* | 0.48 (0.26)* | NA | 0.24 (0.16) | 0.18 (0.15) |
| Have a family member who uses bile (Ref. Does not) | | 2.24 (0.18)*** | 0.69 (0.23)*** | 1.88 (0.26)*** | 1.29 (0.13)*** | 1.06 (0.12)*** |
| Have a friend or colleague who uses bile (Ref. Does not) | | 0.92 (0.2)*** | -0.29 (0.20) | -0.24 (0.17) | 0.58 (0.12)*** | 0.20 (0.12)* |
| ‘Passed’ knowledge test by only selecting true treatment uses for bile (Ref: Did not pass) | | -0.17 (0.18) | -0.81 (0.18)*** | -0.81 (0.16)*** | -0.33 (0.12)*** | -0.24 (0.12)** |
| Thinks wild bile is illegal (Ref: Thinks it is legal or partly legal) | | 0.05 (0.19) | -0.64 (0.18)*** | -0.70 (0.16)*** | 0.08 (0.12) | 0.11 (0.12) |
| Thinks wild bears are decreasing in China (Ref: does not think they are decreasing) | | 0.38 (0.21)* | -0.33 (0.22) | -0.26 (0.20) | 0.13 (0.16) | 0.29 (0.15)* |
| Passive in decisions about mild disease (Ref: Active) | | 0.16 (0.09)* | 0.04 (0.09) | -0.13 (0.08) | 0.05 (0.06) | 0.07 (0.06) |
| Passive in decisions about severe disease (Ref: Active) | | -0.03 (0.08) | -0.01 (0.08) | -0.11 (0.07) | 0.04 (0.05) | -0.04 (0.05) |
| Lives in province with bear farms (Ref: No farms) | | 0.13 (0.17) | 0.02 (0.18) | -0.10 (0.17) | 0.07 (0.12) | -0.01 (0.11) |

9.2 Wild bile user model

9.2.1 Model Selection table

|  | Intercept | Age | CPS mild | CPS severe | gender | Family use | Friend use | Knowledge | Legal | Province farm | Wild bear status | df | logLik | AICc | delta | weight |
| --- | --- | --- | --- | --- | --- | --- | --- | --- | --- | --- | --- | --- | --- | --- | --- | --- |
| 492 | -1.94 | + | -0.14 |  | -0.34 | 1.88 | -0.26 | -0.82 | -0.72 |  |  | 10 | -542.13 | 1104.40 | 0.00 | 0.05 |
| 494 | -1.95 | + |  | -0.12 | -0.35 | 1.87 | -0.26 | -0.80 | -0.73 |  |  | 10 | -542.16 | 1104.50 | 0.05 | 0.05 |
| 496 | -1.81 | + | -0.12 | -0.10 | -0.35 | 1.87 | -0.24 | -0.80 | -0.72 |  |  | 11 | -541.19 | 1104.60 | 0.16 | 0.05 |
| 432 | -1.87 | + | -0.12 | -0.11 | -0.34 | 1.87 |  | -0.80 | -0.73 |  |  | 10 | -542.28 | 1104.70 | 0.30 | 0.04 |
| 428 | -2.01 | + | -0.15 |  | -0.33 | 1.88 |  | -0.82 | -0.72 |  |  | 9 | -543.33 | 1104.80 | 0.36 | 0.04 |
| 430 | -2.02 | + |  | -0.13 | -0.33 | 1.87 |  | -0.79 | -0.74 |  |  | 9 | -543.39 | 1104.90 | 0.49 | 0.04 |
| 1452 | -1.85 | + | -0.15 |  | -0.31 | 1.90 |  | -0.82 | -0.68 |  | -0.28 | 10 | -542.40 | 1105.00 | 0.55 | 0.04 |
| 1456 | -1.72 | + | -0.12 | -0.10 | -0.32 | 1.89 |  | -0.79 | -0.69 |  | -0.27 | 11 | -541.42 | 1105.00 | 0.61 | 0.04 |
| 1516 | -1.81 | + | -0.14 |  | -0.33 | 1.90 | -0.23 | -0.82 | -0.67 |  | -0.24 | 11 | -541.43 | 1105.00 | 0.63 | 0.04 |
| 1454 | -1.87 | + |  | -0.13 | -0.32 | 1.89 |  | -0.79 | -0.69 |  | -0.27 | 10 | -542.51 | 1105.20 | 0.75 | 0.03 |
| 1518 | -1.82 | + |  | -0.12 | -0.33 | 1.89 | -0.24 | -0.79 | -0.69 |  | -0.24 | 11 | -541.49 | 1105.20 | 0.76 | 0.03 |
| 1520 | -1.68 | + | -0.12 | -0.10 | -0.34 | 1.89 | -0.22 | -0.80 | -0.68 |  | -0.24 | 12 | -540.54 | 1105.30 | 0.88 | 0.03 |
| 490 | -2.16 | + |  |  | -0.34 | 1.88 | -0.28 | -0.82 | -0.72 |  |  | 9 | -543.66 | 1105.40 | 1.03 | 0.03 |
| 1004 | -1.88 | + | -0.15 |  | -0.35 | 1.88 | -0.26 | -0.83 | -0.72 | -0.11 |  | 11 | -541.86 | 1105.90 | 1.49 | 0.02 |
| 1514 | -2.02 | + |  |  | -0.32 | 1.90 | -0.25 | -0.82 | -0.68 |  | -0.25 | 10 | -542.93 | 1106.00 | 1.61 | 0.02 |
| 1008 | -1.74 | + | -0.12 | -0.10 | -0.35 | 1.87 | -0.24 | -0.81 | -0.72 | -0.12 |  | 12 | -540.91 | 1106.00 | 1.63 | 0.02 |
| 1006 | -1.90 | + |  | -0.12 | -0.35 | 1.87 | -0.26 | -0.80 | -0.73 | -0.10 |  | 11 | -541.93 | 1106.10 | 1.64 | 0.02 |
| 944 | -1.81 | + | -0.13 | -0.11 | -0.34 | 1.87 |  | -0.80 | -0.73 | -0.12 |  | 11 | -542.00 | 1106.20 | 1.77 | 0.02 |
| 940 | -1.95 | + | -0.16 |  | -0.33 | 1.88 |  | -0.83 | -0.72 | -0.11 |  | 10 | -543.05 | 1106.30 | 1.85 | 0.02 |
| 426 | -2.26 | + |  |  | -0.32 | 1.87 |  | -0.82 | -0.73 |  |  | 8 | -545.09 | 1106.30 | 1.86 | 0.02 |
| 1450 | -2.09 | + |  |  | -0.30 | 1.90 |  | -0.82 | -0.68 |  | -0.28 | 9 | -544.11 | 1106.40 | 1.94 | 0.02 |
| 942 | -1.97 | + |  | -0.13 | -0.34 | 1.87 |  | -0.80 | -0.74 | -0.10 |  | 10 | -543.17 | 1106.50 | 2.09 | 0.02 |
| 1964 | -1.81 | + | -0.15 |  | -0.31 | 1.90 |  | -0.82 | -0.68 | -0.10 | -0.26 | 11 | -542.21 | -542.21 | 1106.60 | 2.20 |
| 1968 | -1.67 | + | -0.13 | -0.10 | -0.32 | 1.89 |  | -0.80 | -0.69 | -0.10 | -0.26 | 12 | -541.22 | -541.22 | 1106.70 | 2.24 |
| 2028 | -1.76 | + | -0.14 |  | -0.33 | 1.90 | -0.23 | -0.82 | -0.68 | -0.10 | -0.23 | 12 | -541.23 | -541.23 | 1106.70 | 2.27 |
| 1444 | -2.03 | + | -0.14 |  |  | 1.90 |  | -0.83 | -0.65 |  | -0.31 | 9 | -544.36 | 1106.80 | 2.43 | 0.02 |
| 2030 | -1.78 | + |  | -0.12 | -0.34 | 1.89 | -0.24 | -0.80 | -0.69 | -0.09 | -0.23 | 12 | -541.33 | -541.33 | 1106.90 | 2.47 |
| 1966 | -1.83 | + |  | -0.13 | -0.32 | 1.89 |  | -0.79 | -0.69 | -0.09 | -0.26 | 11 | -542.36 | -542.36 | 1106.90 | 2.48 |
| 2032 | -1.63 | + | -0.12 | -0.10 | -0.34 | 1.89 | -0.22 | -0.80 | -0.68 | -0.10 | -0.22 | 13 | -540.33 | -540.33 | 1106.90 | 2.50 |
| 1002 | -2.12 | + |  |  | -0.34 | 1.88 | -0.28 | -0.83 | -0.72 | -0.10 |  | 10 | -543.46 | 1107.10 | 2.67 | 0.01 |
| 420 | -2.23 | + | -0.15 |  |  | 1.88 |  | -0.83 | -0.70 |  |  | 8 | -545.51 | 1107.10 | 2.70 | 0.01 |
| 1448 | -1.91 | + | -0.12 | -0.10 |  | 1.90 |  | -0.80 | -0.65 |  | -0.30 | 10 | -543.49 | 1107.10 | 2.72 | 0.01 |
| 484 | -2.17 | + | -0.14 |  |  | 1.88 | -0.23 | -0.83 | -0.69 |  |  | 9 | -544.53 | 1107.20 | 2.77 | 0.01 |
| 1446 | -2.05 | + |  | -0.12 |  | 1.89 |  | -0.80 | -0.66 |  | -0.30 | 9 | -544.53 | 1107.20 | 2.78 | 0.01 |
| 424 | -2.09 | + | -0.12 | -0.10 |  | 1.87 |  | -0.81 | -0.70 |  |  | 9 | -544.58 | 1107.30 | 2.87 | 0.01 |
| 1508 | -2.00 | + | -0.14 |  |  | 1.90 | -0.20 | -0.83 | -0.64 |  | -0.28 | 10 | -543.60 | 1107.40 | 2.94 | 0.01 |
| 486 | -2.18 | + |  | -0.12 |  | 1.87 | -0.23 | -0.81 | -0.70 |  |  | 9 | -544.64 | 1107.40 | 2.98 | 0.01 |
| 422 | -2.24 | + |  | -0.12 |  | 1.87 |  | -0.80 | -0.71 |  |  | 8 | -545.65 | 1107.40 | 2.99 | 0.01 |
| 488 | -2.05 | + | -0.11 | -0.09 |  | 1.87 | -0.22 | -0.81 | -0.70 |  |  | 10 | -543.70 | 1107.60 | 3.14 | 0.01 |
| 1510 | -2.02 | + |  | -0.11 |  | 1.90 | -0.21 | -0.80 | -0.66 |  | -0.27 | 10 | -543.73 | 1107.60 | 3.21 | 0.01 |
| 2026 | -1.99 | + |  |  | -0.32 | 1.90 | -0.26 | -0.82 | -0.68 | -0.08 | -0.24 | 11 | -542.80 | -542.80 | 1107.80 | 3.37 |
| 1512 | -1.88 | + | -0.11 | -0.09 |  | 1.90 | -0.19 | -0.81 | -0.65 |  | -0.27 | 11 | -542.81 | 1107.80 | 3.39 | 0.01 |
| 938 | -2.21 | + |  |  | -0.32 | 1.87 |  | -0.82 | -0.73 | -0.10 |  | 9 | -544.90 | 1107.90 | 3.51 | 0.01 |
| 1442 | -2.26 | + |  |  |  | 1.90 |  | -0.83 | -0.65 |  | -0.31 | 8 | -545.97 | 1108.10 | 3.63 | 0.01 |
| 482 | -2.38 | + |  |  |  | 1.88 | -0.25 | -0.83 | -0.70 |  |  | 8 | -545.99 | 1108.10 | 3.67 | 0.01 |
| 1962 | -2.06 | + |  |  | -0.30 | 1.90 |  | -0.82 | -0.68 | -0.08 | -0.27 | 10 | -543.99 | -543.99 | 1108.10 | 3.72 |
| 1506 | -2.21 | + |  |  |  | 1.91 | -0.23 | -0.83 | -0.65 |  | -0.28 | 9 | -545.03 | 1108.20 | 3.77 | 0.01 |
| 491 | -1.80 |  | -0.15 |  | -0.35 | 1.86 | -0.26 | -0.78 | -0.68 |  |  | 7 | -547.14 | 1108.40 | 3.94 | 0.01 |

9.2.2 Component models (1 = age; 2 = CPS mild; 3 = CPS severe; 4 = gender; 5 = family bile use; 6 = friend bile use; 7 = treatment use knowledge; 8 = legality knowledge; 9 = province with farms; 10 = wild bear status knowledge)

|  | **df** | **logLik** | **AICc** | **delta** | **weight** |
| --- | --- | --- | --- | --- | --- |
| 1/2/4/5/6/7/8 | 10 | -542.13 | 1104.42 | 0 | 0.05 |
| 1/3/4/5/6/7/8 | 10 | -542.15 | 1104.47 | 0.05 | 0.05 |
| 1/2/3/4/5/6/7/8 | 11 | -541.19 | 1104.57 | 0.16 | 0.05 |
| 1/2/3/4/5/7/8 | 10 | -542.28 | 1104.72 | 0.3 | 0.04 |
| 1/2/4/5/7/8 | 9 | -543.33 | 1104.78 | 0.36 | 0.04 |
| 1/3/4/5/7/8 | 9 | -543.39 | 1104.91 | 0.49 | 0.04 |
| 1/2/4/5/7/8/10 | 10 | -542.4 | 1104.96 | 0.55 | 0.04 |
| 1/2/3/4/5/7/8/10 | 11 | -541.42 | 1105.03 | 0.61 | 0.04 |
| 1/2/4/5/6/7/8/10 | 11 | -541.43 | 1105.05 | 0.63 | 0.04 |
| 1/3/4/5/7/8/10 | 10 | -542.51 | 1105.17 | 0.75 | 0.03 |
| 1/3/4/5/6/7/8/10 | 11 | -541.49 | 1105.17 | 0.76 | 0.03 |
| 1/2/3/4/5/6/7/8/10 | 12 | -540.54 | 1105.3 | 0.88 | 0.03 |
| 1/4/5/6/7/8 | 9 | -543.66 | 1105.45 | 1.03 | 0.03 |
| 1/2/4/5/6/7/8/9 | 11 | -541.86 | 1105.9 | 1.49 | 0.02 |
| 1/4/5/6/7/8/10 | 10 | -542.93 | 1106.02 | 1.61 | 0.02 |
| 1/2/3/4/5/6/7/8/9 | 12 | -540.91 | 1106.05 | 1.63 | 0.02 |
| 1/3/4/5/6/7/8/9 | 11 | -541.93 | 1106.06 | 1.64 | 0.02 |
| 1/2/3/4/5/7/8/9 | 11 | -542 | 1106.19 | 1.77 | 0.02 |
| 1/2/4/5/7/8/9 | 10 | -543.05 | 1106.27 | 1.85 | 0.02 |
| 1/4/5/7/8 | 8 | -545.09 | 1106.28 | 1.86 | 0.02 |
| 1/4/5/7/8/10 | 9 | -544.11 | 1106.36 | 1.94 | 0.02 |
| 1/3/4/5/7/8/9 | 10 | -543.17 | 1106.5 | 2.09 | 0.02 |
| 1/2/4/5/7/8/9/10 | 11 | -542.21 | 1106.62 | 2.2 | 0.02 |
| 1/2/3/4/5/7/8/9/10 | 12 | -541.22 | 1106.66 | 2.24 | 0.02 |
| 1/2/4/5/6/7/8/9/10 | 12 | -541.23 | 1106.68 | 2.27 | 0.02 |
| 1/2/5/7/8/10 | 9 | -544.36 | 1106.85 | 2.43 | 0.01 |
| 1/3/4/5/6/7/8/9/10 | 12 | -541.33 | 1106.89 | 2.47 | 0.01 |
| 1/3/4/5/7/8/9/10 | 11 | -542.36 | 1106.9 | 2.48 | 0.01 |
| 1/2/3/4/5/6/7/8/9/10 | 13 | -540.32 | 1106.91 | 2.5 | 0.01 |
| 1/4/5/6/7/8/9 | 10 | -543.46 | 1107.08 | 2.67 | 0.01 |
| 1/2/5/7/8 | 8 | -545.51 | 1107.11 | 2.7 | 0.01 |
| 1/2/3/5/7/8/10 | 10 | -543.49 | 1107.14 | 2.72 | 0.01 |
| 1/2/5/6/7/8 | 9 | -544.53 | 1107.19 | 2.77 | 0.01 |
| 1/3/5/7/8/10 | 9 | -544.53 | 1107.19 | 2.78 | 0.01 |
| 1/2/3/5/7/8 | 9 | -544.58 | 1107.29 | 2.87 | 0.01 |
| 1/2/5/6/7/8/10 | 10 | -543.6 | 1107.35 | 2.94 | 0.01 |
| 1/3/5/6/7/8 | 9 | -544.64 | 1107.4 | 2.98 | 0.01 |
| 1/3/5/7/8 | 8 | -545.65 | 1107.4 | 2.99 | 0.01 |
| 1/2/3/5/6/7/8 | 10 | -543.7 | 1107.56 | 3.14 | 0.01 |
| 1/3/5/6/7/8/10 | 10 | -543.73 | 1107.62 | 3.21 | 0.01 |
| 1/4/5/6/7/8/9/10 | 11 | -542.8 | 1107.79 | 3.37 | 0.01 |
| 1/2/3/5/6/7/8/10 | 11 | -542.81 | 1107.81 | 3.39 | 0.01 |
| 1/4/5/7/8/9 | 9 | -544.9 | 1107.93 | 3.51 | 0.01 |
| 1/5/7/8/10 | 8 | -545.97 | 1108.05 | 3.63 | 0.01 |
| 1/5/6/7/8 | 8 | -545.99 | 1108.09 | 3.67 | 0.01 |
| 1/4/5/7/8/9/10 | 10 | -543.99 | 1108.14 | 3.72 | 0.01 |
| 1/5/6/7/8/10 | 9 | -545.03 | 1108.19 | 3.77 | 0.01 |
| 2/4/5/6/7/8 | 7 | -547.14 | 1108.36 | 3.94 | 0.01 |

9.2.3 Relative variable importance

|  | Family use | knowledge | legal | Age | Gender | CPS_mild | CPS_severe | know_friends | wbstat | province_farm |
| --- | --- | --- | --- | --- | --- | --- | --- | --- | --- | --- |
| Sum | 1 | 1 | 1 | 0.99 | 0.84 | 0.58 | 0.55 | 0.52 | 0.45 | 0.26 |
| N | 48 | 48 | 48 | 47 | 33 | 25 | 24 | 25 | 24 | 16 |

9.3 Higher certainty wild bile user (wild bile +gallbladder/wine) model

9.3.1 Model Selection table

| Model | (Int) | Age | CPS mild | CPS severe | gender | income | Family use | Friend use | knowledge | legal | Province farm | Wild bear status | df | logLik | AICc | delta | weight |
| --- | --- | --- | --- | --- | --- | --- | --- | --- | --- | --- | --- | --- | --- | --- | --- | --- | --- |
| 505 | -1.49 |  |  |  | -0.40 | + | 0.66 | -0.32 | -0.82 | -0.67 |  |  | 11 | -444.77 | 911.70 | 0.00 | 0.08 |
| 1529 | -1.33 |  |  |  | -0.38 | + | 0.69 | -0.29 | -0.81 | -0.61 |  | -0.31 | 12 | -443.87 | 912.00 | 0.23 | 0.07 |
| 1465 | -1.40 |  |  |  | -0.36 | + | 0.70 |  | -0.81 | -0.61 |  | -0.35 | 11 | -444.98 | 912.20 | 0.42 | 0.06 |
| 441 | -1.60 |  |  |  | -0.39 | + | 0.66 |  | -0.82 | -0.68 |  |  | 10 | -446.17 | 912.50 | 0.77 | 0.05 |
| 1449 | -1.38 |  |  |  | -0.40 |  | 0.73 |  | -0.79 | -0.60 |  | -0.34 | 6 | -450.49 | 913.00 | 1.31 | 0.04 |
| 425 | -1.58 |  |  |  | -0.42 |  | 0.70 |  | -0.80 | -0.67 |  |  | 5 | -451.67 | 913.40 | 1.64 | 0.03 |
| 489 | -1.49 |  |  |  | -0.44 |  | 0.71 | -0.26 | -0.80 | -0.66 |  |  | 6 | -450.70 | 913.50 | 1.73 | 0.03 |
| 507 | -1.56 |  | 0.04 |  | -0.40 | + | 0.67 | -0.32 | -0.82 | -0.68 |  |  | 12 | -444.66 | 913.50 | 1.81 | 0.03 |
| 1513 | -1.32 |  |  |  | -0.42 |  | 0.74 | -0.23 | -0.79 | -0.60 |  | -0.31 | 7 | -449.76 | 913.60 | 1.87 | 0.03 |
| 509 | -1.47 |  |  | -0.01 | -0.40 | + | 0.66 | -0.32 | -0.82 | -0.67 |  |  | 12 | -444.76 | 913.80 | 2.02 | 0.03 |
| 1531 | -1.40 |  | 0.05 |  | -0.38 | + | 0.70 | -0.29 | -0.81 | -0.62 |  | -0.31 | 13 | -443.74 | 913.80 | 2.02 | 0.03 |
| 1017 | -1.50 |  |  |  | -0.40 | + | 0.66 | -0.32 | -0.82 | -0.67 | 0.02 |  | 12 | -444.77 | 913.80 | 2.03 | 0.03 |
| 2041 | -1.34 |  |  |  | -0.38 | + | 0.69 | -0.29 | -0.81 | -0.61 | 0.03 | -0.31 | 13 | -443.85 | 914.00 | 2.24 | 0.03 |
| 1533 | -1.32 |  |  | -0.01 | -0.38 | + | 0.69 | -0.29 | -0.81 | -0.61 |  | -0.30 | 13 | -443.87 | 914.00 | 2.26 | 0.03 |
| 1467 | -1.46 |  | 0.04 |  | -0.36 | + | 0.70 |  | -0.81 | -0.62 |  | -0.35 | 12 | -444.91 | 914.00 | 2.30 | 0.02 |
| 1977 | -1.41 |  |  |  | -0.36 | + | 0.70 |  | -0.81 | -0.61 | 0.04 | -0.35 | 12.00 | -444.96 | 914.10 | 2.41 | 0.02 |
| 1469 | -1.37 |  |  | -0.01 | -0.37 | + | 0.69 |  | -0.81 | -0.62 |  | -0.35 | 12.00 | -444.97 | 914.20 | 2.43 | 0.02 |
| 1457 | -1.60 |  |  |  |  | + | 0.71 |  | -0.82 | -0.58 |  | -0.39 | 10.00 | -447.03 | 914.20 | 2.48 | 0.02 |
| 1521 | -1.54 |  |  |  |  | + | 0.71 | -0.26 | -0.82 | -0.58 |  | -0.35 | 11.00 | -446.08 | 914.30 | 2.61 | 0.02 |
| 443 | -1.65 |  | 0.03 |  | -0.39 | + | 0.66 |  | -0.82 | -0.68 |  |  | 11.00 | -446.11 | 914.40 | 2.67 | 0.02 |
| 445 | -1.56 |  |  | -0.02 | -0.39 | + | 0.66 |  | -0.81 | -0.68 |  |  | 11.00 | -446.14 | 914.50 | 2.74 | 0.02 |
| 953 | -1.61 |  |  |  | -0.39 | + | 0.66 |  | -0.82 | -0.68 | 0.02 |  | 11.00 | -446.17 | 914.50 | 2.79 | 0.02 |
| 497 | -1.75 |  |  |  |  | + | 0.67 | -0.30 | -0.83 | -0.64 |  |  | 10.00 | -447.26 | 914.70 | 2.95 | 0.02 |
| 506 | -1.47 | + |  |  | -0.40 | + | 0.65 | -0.34 | -0.81 | -0.67 |  |  | 14.00 | -443.33 | 915.00 | 3.23 | 0.02 |
| 1451 | -1.42 |  | 0.03 |  | -0.40 |  | 0.73 |  | -0.79 | -0.61 |  | -0.34 | 7.00 | -450.45 | 915.00 | 3.25 | 0.02 |
| 1961 | -1.39 |  |  |  | -0.40 |  | 0.73 |  | -0.79 | -0.60 | 0.03 | -0.35 | 7.00 | -450.48 | 915.00 | 3.30 | 0.02 |
| 1530 | -1.30 | + |  |  | -0.38 | + | 0.69 | -0.31 | -0.80 | -0.61 |  | -0.32 | 15.00 | -442.35 | 915.00 | 3.31 | 0.02 |
| 1453 | -1.37 |  |  | -0.01 | -0.40 |  | 0.73 |  | -0.79 | -0.60 |  | -0.34 | 7.00 | -450.49 | 915.10 | 3.32 | 0.02 |
| 433 | -1.84 |  |  |  |  | + | 0.67 |  | -0.82 | -0.65 |  |  | 9.00 | -448.51 | 915.10 | 3.41 | 0.01 |
| 427 | -1.62 |  | 0.02 |  | -0.42 |  | 0.70 |  | -0.80 | -0.67 |  |  | 6.00 | -451.63 | 915.30 | 3.59 | 0.01 |
| 491 | -1.54 |  | 0.03 |  | -0.44 |  | 0.71 | -0.27 | -0.80 | -0.66 |  |  | 7.00 | -450.64 | 915.40 | 3.62 | 0.01 |
| 429 | -1.56 |  |  | -0.01 | -0.43 |  | 0.70 |  | -0.79 | -0.67 |  |  | 6.00 | -451.66 | 915.40 | 3.64 | 0.01 |
| 1450 | -1.38 | + |  |  | -0.40 |  | 0.72 |  | -0.79 | -0.60 |  | -0.37 | 9.00 | -448.63 | 915.40 | 3.65 | 0.01 |
| 937 | -1.58 |  |  |  | -0.42 |  | 0.70 |  | -0.80 | -0.67 | 0.01 |  | 6.00 | -451.67 | 915.40 | 3.66 | 0.01 |
| 493 | -1.49 |  |  | 0.00 | -0.44 |  | 0.71 | -0.26 | -0.80 | -0.66 |  |  | 7.00 | -450.70 | 915.50 | 3.74 | 0.01 |
| 1001 | -1.49 |  |  |  | -0.44 |  | 0.71 | -0.26 | -0.80 | -0.66 | 0.01 |  | 7.00 | -450.70 | 915.50 | 3.75 | 0.01 |
| 1515 | -1.38 |  | 0.03 |  | -0.42 |  | 0.74 | -0.23 | -0.79 | -0.60 |  | -0.31 | 8.00 | -449.69 | 915.50 | 3.76 | 0.01 |
| 511 | -1.54 |  | 0.05 | -0.02 | -0.40 | + | 0.67 | -0.32 | -0.81 | -0.68 |  |  | 13.00 | -444.63 | 915.50 | 3.78 | 0.01 |
| 1466 | -1.37 | + |  |  | -0.36 | + | 0.69 |  | -0.80 | -0.61 |  | -0.36 | 14.00 | -443.61 | 915.50 | 3.80 | 0.01 |
| 1019 | -1.57 |  | 0.04 |  | -0.40 | + | 0.67 | -0.32 | -0.82 | -0.68 | 0.02 |  | 13.00 | -444.65 | 915.60 | 3.83 | 0.01 |
| 2025 | -1.33 |  |  |  | -0.42 |  | 0.73 | -0.23 | -0.79 | -0.60 | 0.03 | -0.31 | 8.00 | -449.75 | 915.60 | 3.87 | 0.01 |
| 1517 | -1.32 |  |  | 0.00 | -0.42 |  | 0.74 | -0.23 | -0.79 | -0.60 |  | -0.31 | 8.00 | -449.76 | 915.60 | 3.89 | 0.01 |
| 1514 | -1.32 | + |  |  | -0.41 |  | 0.72 | -0.25 | -0.79 | -0.60 |  | -0.33 | 10.00 | -447.77 | 915.70 | 3.96 | 0.01 |

9.3.2 Component models (1 = age; 2 = CPS mild; 3 = CPS severe; 4 = gender; 5 = income; 6 = family bile use; 7 = friend bile use; 8 = treatment use knowledge; 9 = legality knowledge; 10 = province with farms; 11 = wild bear status knowledge)

|  | **df** | **logLik** | **AICc** | **delta** | **weight** |
| --- | --- | --- | --- | --- | --- |
| 4/5/6/7/8/9 | 11 | -444.77 | 911.73 | 0 | 0.08 |
| 4/5/6/7/8/9/11 | 12 | -443.87 | 911.97 | 0.23 | 0.07 |
| 4/5/6/8/9/11 | 11 | -444.98 | 912.16 | 0.42 | 0.06 |
| 4/5/6/8/9 | 10 | -446.17 | 912.5 | 0.77 | 0.05 |
| 4/6/8/9/11 | 6 | -450.49 | 913.04 | 1.31 | 0.04 |
| 4/6/8/9 | 5 | -451.67 | 913.38 | 1.64 | 0.03 |
| 4/6/7/8/9 | 6 | -450.7 | 913.46 | 1.73 | 0.03 |
| 2/4/5/6/7/8/9 | 12 | -444.66 | 913.54 | 1.81 | 0.03 |
| 4/6/7/8/9/11 | 7 | -449.76 | 913.61 | 1.87 | 0.03 |
| 3/4/5/6/7/8/9 | 12 | -444.76 | 913.75 | 2.02 | 0.03 |
| 2/4/5/6/7/8/9/11 | 13 | -443.74 | 913.75 | 2.02 | 0.03 |
| 4/5/6/7/8/9/10 | 12 | -444.77 | 913.76 | 2.03 | 0.03 |
| 4/5/6/7/8/9/10/11 | 13 | -443.85 | 913.97 | 2.24 | 0.03 |
| 3/4/5/6/7/8/9/11 | 13 | -443.87 | 914 | 2.26 | 0.02 |
| 2/4/5/6/8/9/11 | 12 | -444.91 | 914.04 | 2.3 | 0.02 |
| 4/5/6/8/9/10/11 | 12 | -444.96 | 914.15 | 2.41 | 0.02 |
| 3/4/5/6/8/9/11 | 12 | -444.97 | 914.16 | 2.43 | 0.02 |
| 5/6/8/9/11 | 10 | -447.03 | 914.21 | 2.48 | 0.02 |
| 5/6/7/8/9/11 | 11 | -446.08 | 914.34 | 2.61 | 0.02 |
| 2/4/5/6/8/9 | 11 | -446.11 | 914.41 | 2.67 | 0.02 |
| 3/4/5/6/8/9 | 11 | -446.14 | 914.48 | 2.74 | 0.02 |
| 4/5/6/8/9/10 | 11 | -446.17 | 914.52 | 2.79 | 0.02 |
| 5/6/7/8/9 | 10 | -447.26 | 914.69 | 2.95 | 0.02 |
| 1/4/5/6/7/8/9 | 14 | -443.33 | 914.97 | 3.23 | 0.02 |
| 2/4/6/8/9/11 | 7 | -450.45 | 914.98 | 3.25 | 0.02 |
| 4/6/8/9/10/11 | 7 | -450.48 | 915.03 | 3.3 | 0.01 |
| 1/4/5/6/7/8/9/11 | 15 | -442.35 | 915.05 | 3.31 | 0.01 |
| 3/4/6/8/9/11 | 7 | -450.49 | 915.06 | 3.32 | 0.01 |
| 5/6/8/9 | 9 | -448.51 | 915.14 | 3.41 | 0.01 |
| 2/4/6/8/9 | 6 | -451.63 | 915.33 | 3.59 | 0.01 |
| 2/4/6/7/8/9 | 7 | -450.64 | 915.35 | 3.62 | 0.01 |
| 3/4/6/8/9 | 6 | -451.66 | 915.38 | 3.64 | 0.01 |
| 1/4/6/8/9/11 | 9 | -448.63 | 915.39 | 3.65 | 0.01 |
| 4/6/8/9/10 | 6 | -451.67 | 915.39 | 3.66 | 0.01 |
| 3/4/6/7/8/9 | 7 | -450.7 | 915.48 | 3.74 | 0.01 |
| 4/6/7/8/9/10 | 7 | -450.7 | 915.48 | 3.75 | 0.01 |
| 2/4/6/7/8/9/11 | 8 | -449.69 | 915.49 | 3.76 | 0.01 |
| 2/3/4/5/6/7/8/9 | 13 | -444.63 | 915.52 | 3.78 | 0.01 |
| 1/4/5/6/8/9/11 | 14 | -443.61 | 915.53 | 3.8 | 0.01 |
| 2/4/5/6/7/8/9/10 | 13 | -444.65 | 915.57 | 3.83 | 0.01 |
| 4/6/7/8/9/10/11 | 8 | -449.75 | 915.6 | 3.87 | 0.01 |
| 3/4/6/7/8/9/11 | 8 | -449.76 | 915.63 | 3.89 | 0.01 |
| 1/4/6/7/8/9/11 | 10 | -447.77 | 915.7 | 3.96 | 0.01 |

9.3.3 Relative variable importance

|  | Family use | knowledge | legal | gender | income | Friend use | Wild bear status | CPS_mild | CPS_severe | Province with a farm | Age |
| --- | --- | --- | --- | --- | --- | --- | --- | --- | --- | --- | --- |
| Sum | 1 | 1 | 1 | 0.93 | 0.69 | 0.54 | 0.52 | 0.18 | 0.16 | 0.16 | 0.06 |
| N | 43 | 43 | 43 | 39 | 25 | 23 | 22 | 10 | 9 | 9 | 5 |

9.4 Farmed bile user model

9.4.1 Model Selection table

|  | **Int** | **Age** | **CPS mild** | **CPS severe** | **gender** | **income** | **Family use** | **Friend use** | **knowledge** | **legality** | **Province with farm** | **Wild bear status** | **df** | **logLik** | **AICc** | **delta** | **weight** |
| --- | --- | --- | --- | --- | --- | --- | --- | --- | --- | --- | --- | --- | --- | --- | --- | --- | --- |
| 250 | -0.48 | + |  |  | -0.46 | + | 1.30 | 0.59 | -0.34 |  |  |  | 13 | -853.90 | 1734.10 | 0 | 0.12 |
| 249 | -0.40 |  |  |  | -0.46 | + | 1.28 | 0.59 | -0.31 |  |  |  | 10 | -857.36 | 1734.90 | 0.83 | 0.08 |
| 1274 | -0.57 | + |  |  | -0.47 | + | 1.29 | 0.58 | -0.34 |  |  | 0.13 | 14 | -853.54 | 1735.40 | 1.33 | 0.06 |
| 254 | -0.55 | + |  | 0.05 | -0.46 | + | 1.30 | 0.58 | -0.35 |  |  |  | 14 | -853.55 | 1735.40 | 1.35 | 0.06 |
| 762 | -0.51 | + |  |  | -0.46 | + | 1.30 | 0.59 | -0.33 |  | 0.08 |  | 14 | -853.69 | 1735.70 | 1.63 | 0.05 |
| 506 | -0.52 | + |  |  | -0.46 | + | 1.30 | 0.59 | -0.34 | 0.07 |  |  | 14 | -853.71 | 1735.70 | 1.68 | 0.05 |
| 1273 | -0.50 |  |  |  | -0.47 | + | 1.28 | 0.58 | -0.32 |  |  | 0.14 | 11 | -856.94 | 1736.10 | 2.01 | 0.04 |
| 252 | -0.49 | + | 0.01 |  | -0.46 | + | 1.30 | 0.59 | -0.34 |  |  |  | 14 | -853.89 | 1736.10 | 2.03 | 0.04 |
| 253 | -0.48 |  |  | 0.04 | -0.46 | + | 1.29 | 0.58 | -0.32 |  |  |  | 11 | -857.02 | 1736.20 | 2.18 | 0.04 |
| 505 | -0.46 |  |  |  | -0.45 | + | 1.29 | 0.58 | -0.32 | 0.09 |  |  | 11 | -857.07 | 1736.30 | 2.27 | 0.04 |
| 761 | -0.43 |  |  |  | -0.46 | + | 1.28 | 0.59 | -0.31 |  | 0.08 |  | 11 | -857.15 | 1736.50 | 2.43 | 0.04 |
| 1278 | -0.64 | + |  | 0.04 | -0.47 | + | 1.30 | 0.58 | -0.35 |  |  | 0.13 | 15 | -853.22 | 1736.80 | 2.73 | 0.03 |
| 251 | -0.40 |  | 0.00 |  | -0.46 | + | 1.28 | 0.59 | -0.31 |  |  |  | 11 | -857.36 | 1736.90 | 2.86 | 0.03 |
| 766 | -0.59 | + |  | 0.05 | -0.46 | + | 1.30 | 0.59 | -0.34 |  | 0.08 |  | 15 | -853.34 | 1737.00 | 2.97 | 0.03 |
| 510 | -0.60 | + |  | 0.05 | -0.46 | + | 1.31 | 0.58 | -0.35 | 0.07 |  |  | 15 | -853.37 | 1737.10 | 3.03 | 0.03 |
| 1786 | -0.60 | + |  |  | -0.47 | + | 1.29 | 0.58 | -0.34 |  | 0.07 | 0.12 | 15 | -853.38 | 1737.10 | 3.05 | 0.03 |
| 1530 | -0.59 | + |  |  | -0.47 | + | 1.30 | 0.58 | -0.35 | 0.05 |  | 0.12 | 15 | -853.46 | 1737.30 | 3.21 | 0.02 |
| 1018 | -0.56 | + |  |  | -0.46 | + | 1.30 | 0.59 | -0.34 | 0.07 | 0.07 |  | 15 | -853.51 | 1737.40 | 3.31 | 0.02 |
| 1276 | -0.58 | + | 0.01 |  | -0.47 | + | 1.29 | 0.58 | -0.34 |  |  | 0.13 | 15 | -853.54 | 1737.40 | 3.37 | 0.02 |
| 256 | -0.55 | + | 0.00 | 0.05 | -0.46 | + | 1.30 | 0.59 | -0.35 |  |  |  | 15 | -853.55 | 1737.40 | 3.39 | 0.02 |
| 1277 | -0.57 |  |  | 0.04 | -0.46 | + | 1.28 | 0.57 | -0.33 |  |  | 0.14 | 12 | -856.62 | 1737.50 | 3.41 | 0.02 |
| 509 | -0.54 |  |  | 0.04 | -0.45 | + | 1.29 | 0.58 | -0.33 | 0.09 |  |  | 12 | -856.73 | 1737.70 | 3.62 | 0.02 |
| 764 | -0.53 | + | 0.01 |  | -0.46 | + | 1.30 | 0.59 | -0.33 |  | 0.08 |  | 15 | -853.67 | 1737.70 | 3.64 | 0.02 |
| 508 | -0.54 | + | 0.01 |  | -0.46 | + | 1.30 | 0.59 | -0.34 | 0.07 |  |  | 15 | -853.71 | 1737.80 | 3.71 | 0.02 |
| 1785 | -0.53 |  |  |  | -0.47 | + | 1.28 | 0.58 | -0.32 |  | 0.07 | 0.14 | 12 | -856.77 | 1737.80 | 3.71 | 0.02 |
| 1529 | -0.53 |  |  |  | -0.46 | + | 1.28 | 0.58 | -0.32 | 0.07 |  | 0.12 | 12 | -856.78 | 1737.80 | 3.73 | 0.02 |
| 765 | -0.51 |  |  | 0.05 | -0.46 | + | 1.29 | 0.58 | -0.32 |  | 0.08 |  | 12 | -856.80 | 1737.80 | 3.76 | 0.02 |
| 1017 | -0.50 |  |  |  | -0.45 | + | 1.29 | 0.59 | -0.32 | 0.09 | 0.08 |  | 12 | -856.86 | 1737.90 | 3.88 | 0.02 |

9.4.2 Component models (1 = age; 2 = CPS mild; 3 = CPS severe; 4 = gender; 5 = income; 6 = family bile use; 7 = friend bile use; 8 = treatment use knowledge; 9 = legality knowledge; 10 = province with farms; 11 = wild bear status knowledge)

|  | **df** | **logLik** | **AICc** | **delta** | **weight** |
| --- | --- | --- | --- | --- | --- |
| 1/4/5/6/7/8 | 13 | -853.89 | 1734.05 | 0 | 0.12 |
| 4/5/6/7/8 | 10 | -857.36 | 1734.88 | 0.83 | 0.08 |
| 1/4/5/6/7/8/11 | 14 | -853.54 | 1735.39 | 1.33 | 0.06 |
| 1/3/4/5/6/7/8 | 14 | -853.55 | 1735.41 | 1.35 | 0.06 |
| 1/4/5/6/7/8/10 | 14 | -853.69 | 1735.69 | 1.63 | 0.05 |
| 1/4/5/6/7/8/9 | 14 | -853.71 | 1735.73 | 1.68 | 0.05 |
| 4/5/6/7/8/11 | 11 | -856.94 | 1736.07 | 2.01 | 0.04 |
| 1/2/4/5/6/7/8 | 14 | -853.89 | 1736.08 | 2.03 | 0.04 |
| 3/4/5/6/7/8 | 11 | -857.02 | 1736.23 | 2.18 | 0.04 |
| 4/5/6/7/8/9 | 11 | -857.07 | 1736.33 | 2.27 | 0.04 |
| 4/5/6/7/8/10 | 11 | -857.14 | 1736.48 | 2.43 | 0.04 |
| 1/3/4/5/6/7/8/11 | 15 | -853.22 | 1736.78 | 2.73 | 0.03 |
| 2/4/5/6/7/8 | 11 | -857.36 | 1736.91 | 2.86 | 0.03 |
| 1/3/4/5/6/7/8/10 | 15 | -853.34 | 1737.03 | 2.97 | 0.03 |
| 1/3/4/5/6/7/8/9 | 15 | -853.37 | 1737.08 | 3.03 | 0.03 |
| 1/4/5/6/7/8/10/11 | 15 | -853.38 | 1737.11 | 3.05 | 0.03 |
| 1/4/5/6/7/8/9/11 | 15 | -853.46 | 1737.26 | 3.21 | 0.02 |
| 1/4/5/6/7/8/9/10 | 15 | -853.51 | 1737.37 | 3.31 | 0.02 |
| 1/2/4/5/6/7/8/11 | 15 | -853.54 | 1737.42 | 3.37 | 0.02 |
| 1/2/3/4/5/6/7/8 | 15 | -853.55 | 1737.45 | 3.39 | 0.02 |
| 3/4/5/6/7/8/11 | 12 | -856.62 | 1737.46 | 3.41 | 0.02 |
| 3/4/5/6/7/8/9 | 12 | -856.73 | 1737.68 | 3.62 | 0.02 |
| 1/2/4/5/6/7/8/10 | 15 | -853.67 | 1737.70 | 3.64 | 0.02 |
| 1/2/4/5/6/7/8/9 | 15 | -853.71 | 1737.76 | 3.71 | 0.02 |
| 4/5/6/7/8/10/11 | 12 | -856.77 | 1737.76 | 3.71 | 0.02 |
| 4/5/6/7/8/9/11 | 12 | -856.78 | 1737.79 | 3.73 | 0.02 |
| 3/4/5/6/7/8/10 | 12 | -856.79 | 1737.82 | 3.76 | 0.02 |
| 4/5/6/7/8/9/10 | 12 | -856.85 | 1737.94 | 3.88 | 0.02 |

9.4.3 Relative variable importance

|  | Gender | Income | Family use | Friend use | knowledge | Age | Wild bear status | CPS severe | Province with farm | Legality | CPS mild |
| --- | --- | --- | --- | --- | --- | --- | --- | --- | --- | --- | --- |
| Sum of weights: | 1 | 1 | 1 | 1 | 1 | 0.62 | 0.26 | 0.26 | 0.24 | 0.24 | 0.15 |
| N containing models: | 28 | 28 | 28 | 28 | 28 | 16 | 9 | 9 | 9 | 9 | 6 |

9.5 Synthetic bile user model

9.5.1 Model Selection table

|  | Int | Age | CPS mild | CPS severe | gender | income | Family use | Friend use | knowledge | legality | Province with farm | Wild bear status | df | logLik | AICc | delta | weight |
| --- | --- | --- | --- | --- | --- | --- | --- | --- | --- | --- | --- | --- | --- | --- | --- | --- | --- |
| 1250 | -0.86 | + |  |  |  |  | 1.06 | 0.20 | -0.25 |  |  | 0.29 | 8 | -910.43 | 1837.00 | 0.00 | 0.07 |
| 1186 | -0.80 | + |  |  |  |  | 1.05 |  | -0.25 |  |  | 0.31 | 7 | -911.84 | 1837.80 | 0.79 | 0.05 |
| 1252 | -0.95 | + | 0.06 |  |  |  | 1.06 | 0.19 | -0.25 |  |  | 0.28 | 9 | -909.88 | 1837.90 | 0.91 | 0.05 |
| 1506 | -0.90 | + |  |  |  |  | 1.06 | 0.20 | -0.26 | 0.10 |  | 0.26 | 9 | -910.09 | 1838.30 | 1.35 | 0.04 |
| 1254 | -0.79 | + |  | -0.04 |  |  | 1.06 | 0.20 | -0.24 |  |  | 0.29 | 9 | -910.15 | 1838.40 | 1.46 | 0.03 |
| 1188 | -0.91 | + | 0.07 |  |  |  | 1.05 |  | -0.25 |  |  | 0.30 | 8 | -911.18 | 1838.50 | 1.49 | 0.03 |
| 226 | -0.64 | + |  |  |  |  | 1.07 | 0.21 | -0.24 |  |  |  | 7 | -912.22 | 1838.50 | 1.55 | 0.03 |
| 1256 | -0.89 | + | 0.07 | -0.05 |  |  | 1.05 | 0.20 | -0.24 |  |  | 0.29 | 10 | -909.37 | 1838.90 | 1.92 | 0.03 |
| 1258 | -0.84 | + |  | -0.03 |  |  | 1.06 | 0.20 | -0.25 |  |  | 0.29 | 9 | -910.40 | 1838.90 | 1.96 | 0.03 |
| 1762 | -0.85 | + |  |  |  |  | 1.06 | 0.20 | -0.25 |  | -0.02 | 0.29 | 9 | -910.42 | 1839.00 | 2.00 | 0.03 |
| 482 | -0.73 | + |  |  |  |  | 1.08 | 0.21 | -0.26 | 0.14 |  |  | 8 | -911.47 | 1839.00 | 2.08 | 0.03 |
| 1442 | -0.85 | + |  |  |  |  | 1.06 |  | -0.26 | 0.10 |  | 0.28 | 8 | -911.49 | 1839.10 | 2.12 | 0.02 |
| 1508 | -0.99 | + | 0.06 |  |  |  | 1.06 | 0.19 | -0.26 | 0.10 |  | 0.26 | 10 | -909.55 | 1839.30 | 2.29 | 0.02 |
| 1190 | -0.75 | + |  |  | -0.03 |  | 1.05 |  | -0.25 |  |  | 0.31 | 8 | -911.63 | 1839.40 | 2.40 | 0.02 |
| 228 | -0.74 | + | 0.06 |  |  |  | 1.07 | 0.21 | -0.24 |  |  |  | 8 | -911.65 | 1839.40 | 2.43 | 0.02 |
| 1266 | -0.83 | + |  |  |  | + | 1.04 | 0.18 | -0.26 |  |  | 0.30 | 13 | -906.66 | 1839.60 | 2.62 | 0.02 |
| 1192 | -0.85 | + | 0.08 | -0.05 |  |  | 1.05 |  | -0.24 |  |  | 0.31 | 9 | -910.74 | 1839.60 | 2.65 | 0.02 |
| 1194 | -0.78 | + |  |  | -0.04 |  | 1.05 |  | -0.25 |  |  | 0.31 | 8 | -911.77 | 1839.70 | 2.68 | 0.02 |
| 1122 | -1.01 | + |  |  |  |  | 1.07 | 0.20 |  |  |  | 0.27 | 7 | -912.83 | 1839.70 | 2.77 | 0.02 |
| 1698 | -0.80 | + |  |  |  |  | 1.05 |  | -0.25 |  | -0.02 | 0.31 | 8 | -911.83 | 1839.80 | 2.78 | 0.02 |
| 1510 | -0.83 | + |  | -0.04 |  |  | 1.06 | 0.20 | -0.25 | 0.10 |  | 0.26 | 10 | -909.81 | 1839.80 | 2.82 | 0.02 |
| 1444 | -0.95 | + | 0.07 |  |  |  | 1.06 |  | -0.26 | 0.10 |  | 0.28 | 9 | -910.84 | 1839.80 | 2.85 | 0.02 |
| 1260 | -0.94 | + | 0.06 |  | -0.03 |  | 1.06 | 0.19 | -0.25 |  |  | 0.29 | 10 | -909.85 | 1839.90 | 2.88 | 0.02 |
| 162 | -0.57 | + |  |  |  |  | 1.06 |  | -0.24 |  |  |  | 6 | -913.90 | 1839.90 | 2.89 | 0.02 |
| 1764 | -0.95 | + | 0.06 |  |  |  | 1.06 | 0.19 | -0.25 |  | -0.01 | 0.28 | 10 | -909.88 | 1839.90 | 2.94 | 0.02 |
| 1202 | -0.78 | + |  |  |  | + | 1.03 |  | -0.26 |  |  | 0.31 | 12 | -907.86 | 1839.90 | 2.96 | 0.02 |
| 484 | -0.83 | + | 0.06 |  |  |  | 1.08 | 0.20 | -0.25 | 0.14 |  |  | 9 | -910.92 | 1840.00 | 3.00 | 0.02 |
| 230 | -0.58 | + |  | -0.04 |  |  | 1.07 | 0.22 | -0.24 |  |  |  | 8 | -911.96 | 1840.00 | 3.05 | 0.02 |
| 1268 | -0.93 | + | 0.07 |  |  | + | 1.03 | 0.17 | -0.26 |  |  | 0.30 | 14 | -905.98 | 1840.30 | 3.29 | 0.01 |
| 1512 | -0.92 | + | 0.07 | -0.05 |  |  | 1.06 | 0.20 | -0.25 | 0.10 |  | 0.26 | 11 | -909.05 | 1840.30 | 3.31 | 0.01 |
| 418 | -0.66 | + |  |  |  |  | 1.07 |  | -0.25 | 0.15 |  |  | 7 | -913.11 | 1840.30 | 3.32 | 0.01 |
| 1514 | -0.89 | + |  |  | -0.02 |  | 1.06 | 0.20 | -0.26 | 0.10 |  | 0.26 | 10 | -910.08 | 1840.30 | 3.34 | 0.01 |
| 2018 | -0.89 | + |  |  |  |  | 1.06 | 0.20 | -0.26 | 0.10 | -0.02 | 0.26 | 10 | -910.08 | 1840.30 | 3.35 | 0.01 |
| 1196 | -0.89 | + | 0.07 |  | -0.04 |  | 1.05 |  | -0.25 |  |  | 0.31 | 9 | -911.12 | 1840.40 | 3.40 | 0.01 |
| 1204 | -0.89 | + | 0.07 |  |  | + | 1.03 |  | -0.26 |  |  | 0.31 | 13 | -907.06 | 1840.40 | 3.41 | 0.01 |
| 1262 | -0.78 | + |  | -0.04 | -0.03 |  | 1.05 | 0.20 | -0.24 |  |  | 0.29 | 10 | -910.11 | 1840.40 | 3.41 | 0.01 |
| 1766 | -0.79 | + |  | -0.04 |  |  | 1.06 | 0.20 | -0.25 |  | -0.02 | 0.29 | 10 | -910.14 | 1840.40 | 3.46 | 0.01 |
| 232 | -0.67 | + | 0.07 | -0.05 |  |  | 1.06 | 0.21 | -0.23 |  |  |  | 9 | -911.16 | 1840.50 | 3.48 | 0.01 |
| 1700 | -0.91 | + | 0.07 |  |  |  | 1.05 |  | -0.25 |  | -0.01 | 0.30 | 9 | -911.18 | 1840.50 | 3.51 | 0.01 |
| 164 | -0.68 | + | 0.07 |  |  |  | 1.06 |  | -0.24 |  |  |  | 7 | -913.21 | 1840.50 | 3.52 | 0.01 |
| 1058 | -0.95 | + |  |  |  |  | 1.07 |  |  |  |  | 0.29 | 6 | -914.23 | 1840.50 | 3.55 | 0.01 |
| 234 | -0.63 | + |  |  | -0.02 |  | 1.07 | 0.21 | -0.24 |  |  |  | 8 | -912.21 | 1840.50 | 3.56 | 0.01 |
| 738 | -0.64 | + |  |  |  |  | 1.07 | 0.21 | -0.24 |  | 0.00 |  | 8 | -912.22 | 1840.50 | 3.57 | 0.01 |
| 486 | -0.67 | + |  | -0.04 |  |  | 1.08 | 0.22 | -0.25 | 0.14 |  |  | 9 | -911.21 | 1840.60 | 3.58 | 0.01 |
| 1124 | -1.10 | + | 0.06 |  |  |  | 1.07 | 0.19 |  |  |  | 0.27 | 8 | -912.26 | 1840.60 | 3.65 | 0.01 |
| 1446 | -0.79 | + |  | -0.03 |  |  | 1.06 |  | -0.25 | 0.10 |  | 0.28 | 9 | -911.29 | 1840.70 | 3.73 | 0.01 |
| 1264 | -0.87 | + | 0.07 | -0.05 | -0.03 |  | 1.05 | 0.19 | -0.24 |  |  | 0.29 | 11 | -909.33 | 1840.90 | 3.88 | 0.01 |
| 1126 | -0.92 | + |  | -0.05 |  |  | 1.07 | 0.20 |  |  |  | 0.28 | 8 | -912.38 | 1840.90 | 3.89 | 0.01 |
| 1768 | -0.88 | + | 0.07 | -0.05 |  |  | 1.05 | 0.20 | -0.24 |  | -0.01 | 0.29 | 11 | -909.37 | 1840.90 | 3.95 | 0.01 |
| 1770 | -0.83 | + |  |  | -0.03 |  | 1.06 | 0.20 | -0.25 |  | -0.02 | 0.29 | 10 | -910.39 | 1840.90 | 3.96 | 0.01 |
| 1270 | -0.76 | + |  | -0.04 |  | + | 1.03 | 0.19 | -0.25 |  |  | 0.30 | 14 | -906.32 | 1840.90 | 3.98 | 0.01 |
| 420 | -0.77 | + | 0.07 |  |  |  | 1.07 |  | -0.25 | 0.15 |  |  | 8 | -912.43 | 1841.00 | 4.00 | 0.01 |

9.5.2 Component models (1 = age; 2 = CPS mild; 3 = CPS severe; 4 = gender; 5 = income; 6 = family bile use; 7 = friend bile use; 8 = treatment use knowledge; 9 = legality knowledge; 10 = province with farms; 11 = wild bear status knowledge)

|  | df | logLik | AICc | delta | weight |
| --- | --- | --- | --- | --- | --- |
| 1/6/7/8/11 | 8 | -910.43 | 1836.97 | 0 | 0.07 |
| 1/6/8/11 | 7 | -911.84 | 1837.76 | 0.79 | 0.05 |
| 1/2/6/7/8/11 | 9 | -909.88 | 1837.89 | 0.91 | 0.04 |
| 1/6/7/8/9/11 | 9 | -910.09 | 1838.32 | 1.35 | 0.04 |
| 1/3/6/7/8/11 | 9 | -910.15 | 1838.43 | 1.46 | 0.03 |
| 1/2/6/8/11 | 8 | -911.18 | 1838.46 | 1.49 | 0.03 |
| 1/6/7/8 | 7 | -912.22 | 1838.52 | 1.55 | 0.03 |
| 1/2/3/6/7/8/11 | 10 | -909.37 | 1838.89 | 1.92 | 0.03 |
| 1/4/6/7/8/11 | 9 | -910.4 | 1838.93 | 1.96 | 0.03 |
| 1/6/7/8/10/11 | 9 | -910.42 | 1838.97 | 2 | 0.03 |
| 1/6/7/8/9 | 8 | -911.47 | 1839.05 | 2.08 | 0.03 |
| 1/6/8/9/11 | 8 | -911.49 | 1839.09 | 2.12 | 0.02 |
| 1/2/6/7/8/9/11 | 10 | -909.55 | 1839.26 | 2.29 | 0.02 |
| 1/3/6/8/11 | 8 | -911.63 | 1839.37 | 2.4 | 0.02 |
| 1/2/6/7/8 | 8 | -911.65 | 1839.4 | 2.43 | 0.02 |
| 1/5/6/7/8/11 | 13 | -906.66 | 1839.59 | 2.62 | 0.02 |
| 1/2/3/6/8/11 | 9 | -910.74 | 1839.62 | 2.65 | 0.02 |
| 1/4/6/8/11 | 8 | -911.77 | 1839.65 | 2.68 | 0.02 |
| 1/6/7/11 | 7 | -912.83 | 1839.74 | 2.77 | 0.02 |
| 1/6/8/10/11 | 8 | -911.83 | 1839.76 | 2.78 | 0.02 |
| 1/3/6/7/8/9/11 | 10 | -909.81 | 1839.79 | 2.82 | 0.02 |
| 1/2/6/8/9/11 | 9 | -910.84 | 1839.82 | 2.85 | 0.02 |
| 1/2/4/6/7/8/11 | 10 | -909.85 | 1839.85 | 2.88 | 0.02 |
| 1/6/8 | 6 | -913.9 | 1839.87 | 2.89 | 0.02 |
| 1/2/6/7/8/10/11 | 10 | -909.88 | 1839.91 | 2.94 | 0.02 |
| 1/5/6/8/11 | 12 | -907.86 | 1839.94 | 2.96 | 0.02 |
| 1/2/6/7/8/9 | 9 | -910.92 | 1839.97 | 3 | 0.02 |
| 1/3/6/7/8 | 8 | -911.96 | 1840.02 | 3.05 | 0.02 |
| 1/2/5/6/7/8/11 | 14 | -905.98 | 1840.27 | 3.29 | 0.01 |
| 1/2/3/6/7/8/9/11 | 11 | -909.05 | 1840.28 | 3.31 | 0.01 |
| 1/6/8/9 | 7 | -913.11 | 1840.29 | 3.32 | 0.01 |
| 1/4/6/7/8/9/11 | 10 | -910.08 | 1840.31 | 3.34 | 0.01 |
| 1/6/7/8/9/10/11 | 10 | -910.08 | 1840.32 | 3.35 | 0.01 |
| 1/2/4/6/8/11 | 9 | -911.12 | 1840.37 | 3.4 | 0.01 |
| 1/2/5/6/8/11 | 13 | -907.06 | 1840.38 | 3.41 | 0.01 |
| 1/3/4/6/7/8/11 | 10 | -910.11 | 1840.39 | 3.41 | 0.01 |
| 1/3/6/7/8/10/11 | 10 | -910.14 | 1840.43 | 3.46 | 0.01 |
| 1/2/3/6/7/8 | 9 | -911.16 | 1840.45 | 3.48 | 0.01 |
| 1/2/6/8/10/11 | 9 | -911.18 | 1840.48 | 3.51 | 0.01 |
| 1/2/6/8 | 7 | -913.21 | 1840.5 | 3.52 | 0.01 |
| 1/6/11 | 6 | -914.23 | 1840.53 | 3.55 | 0.01 |
| 1/4/6/7/8 | 8 | -912.21 | 1840.53 | 3.56 | 0.01 |
| 1/6/7/8/10 | 8 | -912.22 | 1840.55 | 3.57 | 0.01 |
| 1/3/6/7/8/9 | 9 | -911.21 | 1840.55 | 3.58 | 0.01 |
| 1/2/6/7/11 | 8 | -912.26 | 1840.62 | 3.65 | 0.01 |
| 1/3/6/8/9/11 | 9 | -911.29 | 1840.7 | 3.73 | 0.01 |
| 1/2/3/4/6/7/8/11 | 11 | -909.33 | 1840.85 | 3.88 | 0.01 |
| 1/3/6/7/11 | 8 | -912.38 | 1840.86 | 3.89 | 0.01 |
| 1/2/3/6/7/8/10/1 | 1 11 | -909.37 | 1840.92 | 3.95 | 0.01 |
| 1/4/6/7/8/10/11 | 10 | -910.39 | 1840.93 | 3.96 | 0.01 |
| 1/3/5/6/7/8/11 | 14 | -906.32 | 1840.95 | 3.98 | 0.01 |
| 1/2/6/8/9 | 8 | -912.43 | 1840.97 | 4 | 0.01 |

9.5.3 Relative variable importance

|  | Age | Family use | knowledge | Wild bear status | Friend use | CPS mild | CPS severe | legality | Gender | Province with farm | income |
| --- | --- | --- | --- | --- | --- | --- | --- | --- | --- | --- | --- |
| Sum of weights: | 1 | 1 | 0.95 | 0.79 | 0.67 | 0.36 | 0.25 | 0.24 | 0.13 | 0.13 | 0.07 |
| N containing models | 52 | 52 | 48 | 39 | 34 | 21 | 16 | 14 | 9 | 9 | 5 |

9.6 All bile user model

9.6.1 Model Selection table

|  | **Int** | **Age** | **CPS mild** | **CPS severe** | **gender** | **income** | **Family use** | **Friend use** | **knowledge** | **legality** | **Province with farm** | **Wild bear status** | **df** | **logLik** | **AICc** | **delta** | **weight** |
| --- | --- | --- | --- | --- | --- | --- | --- | --- | --- | --- | --- | --- | --- | --- | --- | --- | --- |
| 1147 | 0.09 |  | 0.16 |  | -0.39 | + | 2.22 | 0.90 |  |  |  | 0.3833 | 11 | -463.34 | 948.90 | 0.00 | 0.09 |
| 1275 | 0.20 |  | 0.15 |  | -0.40 | + | 2.21 | 0.91 | -0.17 |  |  | 0.40 | 12 | -462.90 | 950.00 | 1.17 | 0.05 |
| 123 | 0.37 |  | 0.16 |  | -0.37 | + | 2.23 | 0.93 |  |  |  |  | 10 | -465.00 | 950.20 | 1.29 | 0.05 |
| 1145 | 0.34 |  |  |  | -0.40 | + | 2.21 | 0.91 |  |  |  | 0.38 | 10 | -465.05 | 950.30 | 1.40 | 0.04 |
| 1659 | 0.04 |  | 0.17 |  | -0.40 | + | 2.22 | 0.91 |  |  | 0.13 | 0.37 | 12 | -463.05 | 950.30 | 1.45 | 0.04 |
| 1151 | 0.15 |  | 0.16 | -0.04 | -0.39 | + | 2.22 | 0.90 |  |  |  | 0.38 | 12 | -463.24 | 950.70 | 1.84 | 0.03 |
| 1131 | 0.06 |  | 0.15 |  | -0.41 |  | 2.23 | 0.92 |  |  |  | 0.38 | 6 | -469.34 | 950.70 | 1.88 | 0.03 |
| 1403 | 0.08 |  | 0.16 |  | -0.39 | + | 2.22 | 0.90 |  | 0.03 |  | 0.37 | 12 | -463.32 | 950.90 | 2.00 | 0.03 |
| 1148 | 0.02 | + | 0.16 |  | -0.41 | + | 2.24 | 0.92 |  |  |  | 0.37 | 14 | -461.30 | 950.90 | 2.05 | 0.03 |
| 1273 | 0.45 |  |  |  | -0.40 | + | 2.20 | 0.92 | -0.18 |  |  | 0.40 | 11 | -464.53 | 951.20 | 2.37 | 0.03 |
| 1132 | -0.04 | + | 0.16 |  | -0.42 |  | 2.25 | 0.94 |  |  |  | 0.37 | 9 | -466.58 | 951.30 | 2.42 | 0.03 |
| 635 | 0.29 |  | 0.17 |  | -0.38 | + | 2.23 | 0.93 |  |  | 0.15 |  | 11 | -464.60 | 951.40 | 2.52 | 0.02 |
| 251 | 0.47 |  | 0.15 |  | -0.37 | + | 2.22 | 0.94 | -0.14 |  |  |  | 11 | -464.67 | 951.50 | 2.67 | 0.02 |
| 1787 | 0.14 |  | 0.16 |  | -0.40 | + | 2.21 | 0.91 | -0.16 |  | 0.12 | 0.38 | 13 | -462.64 | 951.60 | 2.68 | 0.02 |
| 121 | 0.62 |  |  |  | -0.37 | + | 2.23 | 0.94 |  |  |  |  | 9 | -466.71 | 951.60 | 2.69 | 0.02 |
| 1259 | 0.17 |  | 0.14 |  | -0.41 |  | 2.22 | 0.93 | -0.18 |  |  | 0.40 | 7 | -468.82 | 951.70 | 2.85 | 0.02 |
| 379 | 0.28 |  | 0.16 |  | -0.37 | + | 2.24 | 0.93 |  | 0.12 |  |  | 11 | -464.77 | 951.70 | 2.87 | 0.02 |
| 1139 | -0.13 |  | 0.16 |  |  | + | 2.21 | 0.90 |  |  |  | 0.35 | 10 | -465.81 | 951.80 | 2.92 | 0.02 |
| 1276 | 0.14 | + | 0.16 |  | -0.42 | + | 2.23 | 0.93 | -0.19 |  |  | 0.39 | 15 | -460.77 | 951.90 | 3.01 | 0.02 |
| 1129 | 0.29 |  |  |  | -0.41 |  | 2.22 | 0.93 |  |  |  | 0.38 | 5 | -470.94 | 951.90 | 3.05 | 0.02 |
| 1260 | 0.09 | + | 0.15 |  | -0.42 |  | 2.24 | 0.95 | -0.21 |  |  | 0.38 | 10 | -465.89 | 951.90 | 3.06 | 0.02 |
| 1279 | 0.24 |  | 0.16 | -0.03 | -0.40 | + | 2.21 | 0.91 | -0.16 |  |  | 0.40 | 13 | -462.84 | 951.90 | 3.08 | 0.02 |
| 127 | 0.42 |  | 0.16 | -0.03 | -0.37 | + | 2.23 | 0.93 |  |  |  |  | 11 | -464.91 | 952.00 | 3.14 | 0.02 |
| 124 | 0.29 | + | 0.16 |  | -0.39 | + | 2.26 | 0.95 |  |  |  |  | 13 | -462.88 | 952.00 | 3.15 | 0.02 |
| 1531 | 0.18 |  | 0.15 |  | -0.40 | + | 2.21 | 0.91 | -0.17 | 0.04 |  | 0.38 | 13 | -462.88 | 952.00 | 3.15 | 0.02 |
| 1657 | 0.31 |  |  |  | -0.40 | + | 2.22 | 0.92 |  |  | 0.09 | 0.37 | 11 | -464.92 | 952.00 | 3.16 | 0.02 |
| 107 | 0.34 |  | 0.15 |  | -0.38 |  | 2.24 | 0.95 |  |  |  |  | 5 | -471.03 | 952.10 | 3.23 | 0.02 |
| 1663 | 0.09 |  | 0.17 | -0.04 | -0.40 | + | 2.22 | 0.91 |  |  | 0.13 | 0.37 | 13 | -462.95 | 952.20 | 3.29 | 0.02 |
| 1401 | 0.33 |  |  |  | -0.39 | + | 2.22 | 0.91 |  | 0.02 |  | 0.38 | 11 | -465.05 | 952.30 | 3.42 | 0.02 |
| 1149 | 0.35 |  |  | -0.01 | -0.40 | + | 2.21 | 0.91 |  |  |  | 0.38 | 11 | -465.05 | 952.30 | 3.42 | 0.02 |
| 1915 | 0.02 |  | 0.17 |  | -0.40 | + | 2.22 | 0.91 |  | 0.04 | 0.13 | 0.36 | 13 | -463.03 | 952.30 | 3.45 | 0.02 |
| 1643 | 0.01 |  | 0.16 |  | -0.41 |  | 2.23 | 0.93 |  |  | 0.11 | 0.37 | 7 | -469.14 | 952.40 | 3.48 | 0.02 |
| 108 | 0.22 | + | 0.16 |  | -0.40 |  | 2.26 | 0.96 |  |  |  |  | 8 | -468.13 | 952.40 | 3.49 | 0.02 |
| 1660 | -0.03 | + | 0.17 |  | -0.42 | + | 2.24 | 0.93 |  |  | 0.12 | 0.36 | 15 | -461.06 | 952.50 | 3.60 | 0.01 |
| 1146 | 0.28 | + |  |  | -0.41 | + | 2.24 | 0.93 |  |  |  | 0.38 | 13 | -463.12 | 952.50 | 3.64 | 0.01 |
| 115 | 0.14 |  | 0.16 |  |  | + | 2.22 | 0.93 |  |  |  |  | 9 | -467.22 | 952.60 | 3.70 | 0.01 |
| 1135 | 0.10 |  | 0.16 | -0.03 | -0.41 |  | 2.23 | 0.93 |  |  |  | 0.38 | 7 | -469.29 | 952.70 | 3.78 | 0.01 |
| 1152 | 0.08 | + | 0.17 | -0.04 | -0.41 | + | 2.24 | 0.93 |  |  |  | 0.38 | 15 | -461.17 | 952.70 | 3.81 | 0.01 |
| 1257 | 0.41 |  |  |  | -0.41 |  | 2.21 | 0.94 | -0.20 |  |  | 0.40 | 6 | -470.32 | 952.70 | 3.83 | 0.01 |
| 1407 | 0.13 |  | 0.17 | -0.04 | -0.39 | + | 2.22 | 0.90 |  | 0.03 |  | 0.37 | 13 | -463.22 | 952.70 | 3.84 | 0.01 |
| 1387 | 0.04 |  | 0.15 |  | -0.40 |  | 2.23 | 0.92 |  | 0.04 |  | 0.37 | 7 | -469.32 | 952.70 | 3.85 | 0.01 |
| 249 | 0.72 |  |  |  | -0.38 | + | 2.22 | 0.95 | -0.16 |  |  |  | 10 | -466.31 | 952.80 | 3.90 | 0.01 |
| 763 | 0.39 |  | 0.16 |  | -0.38 | + | 2.23 | 0.94 | -0.14 |  | 0.15 |  | 12 | -464.30 | 952.80 | 3.96 | 0.01 |
| 1130 | 0.21 | + |  |  | -0.42 |  | 2.24 | 0.94 |  |  |  | 0.37 | 8 | -468.36 | 952.80 | 3.96 | 0.01 |

9.6.2 Component models (1 = age; 2 = CPS mild; 3 = CPS severe; 4 = gender; 5 = income; 6 = family bile use; 7 = friend bile use; 8 = treatment use knowledge; 9 = legality knowledge; 10 = province with farms; 11 = wild bear status knowledge)

|  | **df** | **logLik** | **AICc** | **delta** | **weight** |
| --- | --- | --- | --- | --- | --- |
| 2/4/5/6/7/11 | 11 | -463.34 | 948.87 | 0 | 0.09 |
| 2/4/5/6/7/8/11 | 12 | -462.9 | 950.03 | 1.17 | 0.05 |
| 2/4/5/6/7 | 10 | -465 | 950.16 | 1.29 | 0.05 |
| 4/5/6/7/11 | 10 | -465.05 | 950.27 | 1.4 | 0.04 |
| 2/4/5/6/7/10/11 | 12 | -463.05 | 950.32 | 1.45 | 0.04 |
| 2/3/4/5/6/7/11 | 12 | -463.24 | 950.7 | 1.84 | 0.03 |
| 2/4/6/7/11 | 6 | -469.34 | 950.74 | 1.88 | 0.03 |
| 2/4/5/6/7/9/11 | 12 | -463.32 | 950.87 | 2 | 0.03 |
| 1/2/4/5/6/7/11 | 14 | -461.3 | 950.91 | 2.05 | 0.03 |
| 4/5/6/7/8/11 | 11 | -464.52 | 951.24 | 2.37 | 0.03 |
| 1/2/4/6/7/11 | 9 | -466.58 | 951.29 | 2.42 | 0.03 |
| 2/4/5/6/7/10 | 11 | -464.6 | 951.39 | 2.52 | 0.02 |
| 2/4/5/6/7/8 | 11 | -464.67 | 951.54 | 2.67 | 0.02 |
| 2/4/5/6/7/8/10/11 | 13 | -462.64 | 951.55 | 2.68 | 0.02 |
| 4/5/6/7 | 9 | -466.71 | 951.56 | 2.69 | 0.02 |
| 2/4/6/7/8/11 | 7 | -468.82 | 951.72 | 2.85 | 0.02 |
| 2/4/5/6/7/9 | 11 | -464.77 | 951.74 | 2.87 | 0.02 |
| 2/5/6/7/11 | 10 | -465.81 | 951.79 | 2.92 | 0.02 |
| 1/2/4/5/6/7/8/11 | 15 | -460.76 | 951.88 | 3.01 | 0.02 |
| 4/6/7/11 | 5 | -470.94 | 951.92 | 3.05 | 0.02 |
| 1/2/4/6/7/8/11 | 10 | -465.88 | 951.93 | 3.06 | 0.02 |
| 2/3/4/5/6/7/8/11 | 13 | -462.84 | 951.94 | 3.08 | 0.02 |
| 2/3/4/5/6/7 | 11 | -464.91 | 952.01 | 3.14 | 0.02 |
| 1/2/4/5/6/7 | 13 | -462.87 | 952.01 | 3.15 | 0.02 |
| 2/4/5/6/7/8/9/11 | 13 | -462.88 | 952.02 | 3.15 | 0.02 |
| 4/5/6/7/10/11 | 11 | -464.92 | 952.02 | 3.16 | 0.02 |
| 2/4/6/7 | 5 | -471.03 | 952.1 | 3.23 | 0.02 |
| 2/3/4/5/6/7/10/11 | 13 | -462.95 | 952.15 | 3.29 | 0.02 |
| 4/5/6/7/9/11 | 11 | -465.05 | 952.29 | 3.42 | 0.02 |
| 3/4/5/6/7/11 | 11 | -465.05 | 952.29 | 3.42 | 0.02 |
| 2/4/5/6/7/9/10/11 | 13 | -463.03 | 952.32 | 3.45 | 0.02 |
| 2/4/6/7/10/11 | 7 | -469.13 | 952.35 | 3.48 | 0.02 |
| 1/2/4/6/7 | 8 | -468.13 | 952.36 | 3.49 | 0.02 |
| 1/2/4/5/6/7/10/11 | 15 | -461.06 | 952.46 | 3.6 | 0.01 |
| 1/4/5/6/7/11 | 13 | -463.12 | 952.51 | 3.64 | 0.01 |
| 2/5/6/7 | 9 | -467.22 | 952.57 | 3.7 | 0.01 |
| 2/3/4/6/7/11 | 7 | -469.29 | 952.65 | 3.78 | 0.01 |
| 1/2/3/4/5/6/7/11 | 15 | -461.17 | 952.68 | 3.81 | 0.01 |
| 4/6/7/8/11 | 6 | -470.32 | 952.7 | 3.83 | 0.01 |
| 2/3/4/5/6/7/9/11 | 13 | -463.22 | 952.71 | 3.84 | 0.01 |
| 2/4/6/7/9/11 | 7 | -469.32 | 952.72 | 3.85 | 0.01 |
| 4/5/6/7/8 | 10 | -466.31 | 952.77 | 3.9 | 0.01 |
| 2/4/5/6/7/8/10 | 12 | -464.3 | 952.83 | 3.96 | 0.01 |
| 1/4/6/7/11 | 8 | -468.36 | 952.83 | 3.96 | 0.01 |

9.6.3 Relative variable importance

|  | Family use | Friend use | Gender | CPS mild | Income | Wild bear status | knowledge | Age | Province with farm | CPS severe | legal |
| --- | --- | --- | --- | --- | --- | --- | --- | --- | --- | --- | --- |
| Sum of weights: | 1 | 1 | 0.97 | 0.79 | 0.78 | 0.76 | 0.25 | 0.18 | 0.18 | 0.14 | 0.13 |
| N containing models | 44 | 44 | 42 | 33 | 32 | 32 | 12 | 10 | 9 | 8 | 7 |

***S10. The Random Parameter Logit model for the DCE***

For the DCE we estimated a random parameters logit model (RPL) using the R package ‘Apollo’ (Hess 2019 a,b) to examine broad preferences across the whole sample, considering individual heterogeneity.

RPL model estimates and standard deviations. Shading denotes significance at least p <0.05 with positive coefficients in yellow, negative in grey. Significance: * = p <0.1, ** = p <0.05, *** p <0.01

| ***Attribute*** | ***Level*** | ***Mean effect on choice (SE)*** | ***St. Deviation (SE)*** |
| --- | --- | --- | --- |
| *Type (base: wild)* | *Farmed* | 0.2 (0.1) *** | -1.1 (0.1) *** |
|  | *Synthetic* | 1.1 (0.1) *** | -2.3 (0.1) *** |
| *Place of purchase (base: pharmacy)* | *Online* | -1.1 (0.1) *** | -1.6 (0.1) *** |
|  | *Personal contact* | -1.6 (0.1) *** | -1.1 (0.1) *** |
|  | *TCM Market* | -0.6 (0.1) *** | 0.1 (0.2) |
|  | *Hospital* | 0.9 (0.1) *** | -1.5 (0.1) *** |
|  | *Bear farm* | -1.0 (0.1) *** | -0.9 (0.1) *** |
| *Product form (base: gallbladder)* | *Medicinal wine* | -0.7 (0.1) *** | -0.1 (0.3) |
|  | *Powder* | -0.7 (0.1) *** | -0.04 (0.2) |
|  | *Liquids* | -0.4 (0.1) *** | 0.5 (0.2) *** |
|  | *Tablets/capsules* | -0.3 (0.1) *** | -0.3 (0.1) *** |
|  | *Tea* | -0.8 (0.1) *** | -0.8 (0.1) *** |
| *ASC* |  | -6.1 (0.4) *** | -4.9 (0.4) *** |
| *Price* |  | -0.001 (0.000)*** |  |
| **Interactions** |  |  |  |
| *Early Liver * Price* |  | 0.0004 (0.000) *** |  |
| Early Liver * Farmed |  | 0.3 (0.1) *** |  |
| Early Liver * Synthetic |  | -0.5 (0.2) *** |  |
| *Late liver * Price* |  | 0.003 (0.0002)*** |  |
| Late Liver * Farmed |  | 0.7 (0.1) *** |  |
| Late Liver * Synthetic |  | -0.6 (0.2) *** |  |

***S11. Latent class estimation and model selection***

Latent class models can be estimated in Latent Class Gold with either dummy or effects

codding. With dummy coding, the class membership model uses one class as the reference

class such that parameters are fixed to zero for Class 1, and parameters for Classes 2-5 are

interpreted as differences from the reference class. Effects coding estimates class

membership parameters for all classes and parameters across all five classes sum to zero.

With effects coding, parameters are compared to the sample average rather than reference

class as with dummy coding (Vermunt & Magidson 2016).

There is no correct way to select the number of classes in a LCM. In practice, it is typical to choose the model with the lowest BIC. However, increasing the number of classes often results in lower BIC, while only explaining a smaller and smaller percentage of the sample. Therefore, it is common to stop increasing the number of classes, when doing so leads to class sizes is less than 5%. From Table S, the BIC is minimised at the 7-class model, but as we increase classes past 5, we get very small class sizes (< 3%).

Table 11.1. Model selection using BIC for our LCM

|  | LL | BIC(LL) |
| --- | --- | --- |
| 1-Class Choice | -11420.82 | 22981.86 |
| 2-Class Choice | -10151.32 | 20625.16 |
| 3-Class Choice | -9689.33 | 19883.47 |
| 4-Class Choice | -9436.44 | 19559.99 |
| 5-Class Choice | -9250.73 | 19370.84 |
| 6-Class Choice | -9088.59 | 19228.86 |
| 7-Class Choice | -8974.90 | 19183.78 |
| 8-Class Choice | -8888.74 | 19193.74 |
| 9-Class Choice | -8813.37 | 19225.29 |
| 10-Class Choice | -8751.78 | 19284.41 |

***S12 Descriptive statistics of respondents in each LCM class***

| Covariate | Percentage in each class | | | | |
| --- | --- | --- | --- | --- | --- |
|  | **Class 1** | **Class 2** | **Class 3** | **Class 4** | **Class 5** |
| Male = 1 | 31.9% | 27.9% | 25.2% | 8.5% | 6.5% |
| Have used farmed bile = 1 | 34.6% | 37.6% | 23.1% | 2.3% | 2.5% |
| Have used wild bile = 1 | 14.8% | 58.5% | 23.4% | 0.9% | 2.6% |
| Have family who use bile = 1 | 33.8% | 35.8% | 23.1% | 4.6% | 2.7% |
| Know bear bile is illegal = 1 | 40.9% | 23.0% | 19.9% | 10.4% | 5.8% |

***S13. Results of the 5-class LC model for the DCE.***

Shading denotes significance at least p <0.05 with positive coefficients in yellow and negative in grey.

|  |  | Class 1 | Class 2 | Class 3 | Class 4 | Class 5 |
| --- | --- | --- | --- | --- | --- | --- |
| Type (Ref: wild) | Farmed | 1.41 (0.21) | -0.5 (0.14) | 0.39 (0.31) | 1.48 (0.77) | -0.69 (0.5) |
|  | Synthetic | 3.28 (0.29) | -0.57 (0.2) | -2.33 (0.48) | 4.91 (0.85) | -7.31 (9.5) |
| Place (Ref: Pharmacy) | Online | -1.46 (0.12) | -0.8 (0.09) | -4.09 (0.28) | -0.4 (0.4) | -1.12 (0.28) |
|  | Personal Contact | -1.67 (0.13) | -0.34 (0.09) | -4.65 (0.37) | -3.7 (0.74) | -1.54 (0.41) |
|  | TCM market | -1.12 (0.13) | -0.03 (0.08) | -2.47 (0.25) | -0.65 (0.38) | -0.94 (0.34) |
|  | Hospital | 0.87 (0.14) | 0.17 (0.1) | 0.62 (0.21) | -1.15 (0.44) | 0.83 (0.31) |
|  | Bear farm | -0.82 (0.11) | -0.19 (0.1) | -2.82 (0.24) | -1.79 (0.39) | -1.49 (0.43) |
| Form (Ref: Gallbladder) | Medicinal Wine | -1.09 (0.21) | -0.48 (0.12) | 1.33 (0.61) | -0.26 (0.94) | 0.82 (0.48) |
|  | Powder | -0.3 (0.21) | -0.68 (0.15) | 2.11 (0.66) | 1.11 (0.87) | 1.37 (0.58) |
|  | Liquids | 0.11 (0.28) | -0.58 (0.16) | 2.79 (0.79) | 2.88 (1.27) | 1.76 (0.78) |
|  | Tablets/Capsules | -0.07 (0.27) | -0.41 (0.17) | 3.49 (0.76) | 1.44 (1.17) | 1.57 (0.77) |
|  | Tea | -0.63 (0.28) | -0.66 (0.17) | 2.62 (0.72) | 1.37 (1.18) | 1.88 (0.74) |
| Price | | -0.0007  (0.0001) | -0.0005  (0.0001) | -0.0001  (0.0002) | -0.0001  (0.0003) | 0.0001  (0.0002) |
| ASC | | -3 (0.3) | -5 (0.4) | -3 (0.8) | 4 (1) | 3 (0.7) |
| Interactions | Early Liver * Price | 0.00 (0) | 0.00 (0) | 0.00 (0) | 0 (0) | 0 (0) |
|  | Late Liver * Price | 0.00 (0) | 0.00 (0) | 0.01 (0) | 0.00 (0) | 0.00 (0) |
|  | Early Liver * Farmed | 0.54 (0.17) | 0.28 (0.17) | 0.68 (0.28) | 0.97 (0.54) | 0.85 (0.41) |
|  | Late Liver * Farmed | -0.12 (0.30) | 0.94 (0.32) | -0.88 (0.73) | -0.14 (0.56) | 0.58 (0.37) |
|  | Early Liver * Synthetic | -0.94 (0.31) | -0.22 (0.25) | 2.61 (0.55) | 1.18 (0.73) | 7.43 (9.5) |
|  | Late Liver * Synthetic | -1.78 (0.37) | 0.52 (0.37) | 0.81 (0.8) | 0.53 (0.7) | 7.02 (9.5) |
| Covariates | Gender | -0.65 (0.23) | -0.64 (0.25) | 0.07 (0.26) | -0.06 (0.36) | 1.28 (0.45) |
|  | Farmed user | 0.9 (0.25) | 1.49 (0.27) | 0.9 (0.28) | -2.32 (0.46) | -0.97 (0.47) |
|  | Family bile use | 0.34 (0.13) | 0.87 (0.15) | 0.31 (0.14) | -0.57 (0.18) | -0.94 (0.23) |
|  | Think wild bile is legal (Ref: does not) | 0.38 (0.14) | -0.84 (0.13) | -0.54 (0.14) | 0.78 (0.22) | 0.22 (0.23) |
|  | Wild bile user | -1.43 (0.44) | 1.42 (0.36) | 0.4 (0.39) | -1.27 (0.86) | 0.88 (0.69) |

***S14. Willingness to Pay estimates for both Latent Class Model classes with significant price attributes*** *(law-abiding consumers* and *experienced* *switchers)*, with numbers representing the value in CNY that respondents in a class are prepared to pay for a course of treatment with different levels of the attributes when compared to the base level. Shading denotes significance at least p <0.05 with positive coefficients in yellow and negative in grey.

| **Attribute** | **Level** | **Willingness to Pay in CNY (SE)** | |
| --- | --- | --- | --- |
|  |  | **Law-abiding consumers** | **Experienced switchers** |
| *Type (Ref: wild)* | *Farmed* | 1818 (424) | -903 (207) |
|  | *Synthetic* | 4430 (743) | -1020 (317) |
| *Place of purchase (Ref: pharmacy)* | *Online* | -1993 (283) | -1482 (197) |
|  | *Personal contact* | -2276 (337) | -630 (171) |
|  | *TCM Market* | -1540 (257) | -36 (346) |
|  | *Hospital* | 1215 (260) | 308 (193) |
|  | *Bear farm* | -1108 (199) | -366 (185) |
| *Product form (Ref: gallbladder)* | *Medicinal wine* | -1490 (305) | -882 (208) |
|  | *Powder* | -447 (364) | -1262 (199) |
|  | *Liquids* | 98 (400) | -1102 (262) |
|  | *Tablets or capsules* | -162 (359) | -761 (267) |
|  | *Tea* | -939 (339) | -1215 (271) |
| *ASC* |  | -4135 (452) | -9732 (1153) |

**References**

Bybee, S., Cloyes, K., Baucom, B., Supiano, K., Mooney, K. and Ellington, L., 2021. Bots and nots: Safeguarding online survey research with underrepresented and diverse populations. Psychology & Sexuality.

Hess, S. & Palma, D. (2019a), Apollo: a flexible, powerful and customisable freeware package for choice model estimation and application, Journal of Choice Modelling

Hess, S. & Palma, D. (2019b), Apollo v.0.1.0,[www.ApolloChoiceModelling.com](http://www.ApolloChoiceModelling.com)

Vermunt, J. K., & Magidson, J. (2016). Technical Guide for Latent GOLD 5.1: Basic, Advanced, and Syntax. Retrieved from http://www.statisticalinnovations.com
